# 3D-printed porous titanium versus PEEK interbody cages in lumbar fusion: biomechanical rationale, clinical evidence, and emerging indications

**DOI:** 10.3389/fbioe.2026.1952166

**Published:** 2026-09-28

**Authors:** Dongfang Yang, Yutong Hu, Hao Fu, Mingyang Zhu, Haibin Zhang, Mei Li

**Affiliations:** 1 Department of Orthopedics, The Second Affiliated Hospital of Naval Medical University (Shanghai Changzheng Hospital), Shanghai, China; 2 Department of Orthopedics, The Second Affiliated Hospital of Soochow University, Suzhou, Jiangsu, China; 3 Department of Orthopedics, Zibo Municipal Hospital, Zibo, Shandong, China; 4 Department of Orthopedics, The Second Affiliated Hospital of Inner Mongolia Medical University, Hohhot, China; 5 Department of Neurosurgery, Central Hospital of Dalian University of Technology (Dalian Municipal Central Hospital), Dalian, China

**Keywords:** 3D printing, biomechanics, lumbar interbody fusion, osseointegration, polyetheretherketone (PEEK), porous titanium, revision, subsidence

## Abstract

Lumbar interbody fusion is a cornerstone treatment for degenerative spinal conditions. PEEK cages offer an elastic modulus approximating cancellous bone and radiolucency, yet their bioinert surface can result in fibrous encapsulation rather than direct bone apposition. 3D-printed porous titanium (3D-pTi) cages address these limitations through an engineered porous architecture that reduces effective stiffness to near-cancellous bone levels while enabling bone ingrowth throughout the implant volume. This narrative review synthesizes evidence spanning *in vitro* surface science, large-animal models, human retrieval histology, randomized trials, cohort studies, and meta-analyses. A rat tibial experiment demonstrated that surface topography accounted for substantially more variance in fixation strength than surface chemistry under the specific conditions tested (∼72 μm pore size, 8-week endpoint); whether this relative weighting extrapolates to the human lumbar interbody environment has not been established. Large-animal studies show that 3D-pTi cages without bone graft can outperform grafted PEEK cages, and human retrieval histology confirms lamellar bone in direct contact with the titanium scaffold without fibrous interposition. A randomized trial terminated at interim analysis reported 6-month fusion rates of 100% for 3D-pTi versus 0% for PEEK, though small sample size limits generalizability. Meta-analytic evidence consistently suggests accelerated early fusion with 3D-pTi, though between-group differences narrow by 12 months and no network meta-analysis pairwise comparison reached statistical significance. The clinical significance of accelerated fusion for long-term patient-reported outcomes remains unestablished—meta-analyses report no significant between-group differences in pain or disability scores. Regression analyses indicate that cage surface area and shape, rather than material identity, independently predict subsidence. Titanium-coated PEEK improves early fusion over uncoated PEEK but cannot achieve three-dimensional bone ingrowth. PEEK retains specific advantages: radiolucency, a well-characterized safety record, and greater ease of revision when cage removal is anticipated. For most clinical scenarios, current evidence does not mandate one material; cage selection should be individualized.

## 1 Introduction

Lumbar degenerative diseases—including degenerative disc disease, lumbar spinal stenosis, and degenerative spondylolisthesis—represent the most common indications for spinal surgery in aging populations worldwide. Epidemiological data indicate that lumbar degenerative disease affects an estimated 27.3% of individuals aged 65 years or older, with a disproportionate burden in women (34.7%) compared to men (18.1%) (Duan et al., 2024). In the United States, lumbar fusion procedure rates increased by 134% between 2004 and 2015, with the greatest growth observed among patients over 65 years of age (Martin et al., 2019). For patients with radiographically confirmed segmental instability, significant neural compression refractory to conservative management, or progressive deformity, lumbar interbody fusion (LIF) remains an established surgical strategy to restore spinal stability, achieve neural decompression, and correct sagittal malalignment (Reid et al., 2019).

The interbody cage is the central structural element in LIF, serving the triple functions of restoring intervertebral disc and foraminal height, providing anterior column mechanical support, and acting as an osteoconductive scaffold for interbody bone formation (Mobbs et al., 2015). Since the pioneering work of Bagby and Kuslich in the 1990s, the material composition of interbody cages has undergone progressive evolution—from stainless steel and solid titanium, through cortical allograft and carbon-fiber-reinforced polymers, to the current dominance of polyetheretherketone (PEEK) (Muthiah et al., 2022; Enache et al., 2026). PEEK was introduced to spine surgery in the late 1990s and rapidly became the most widely used interbody material. Its appeal lies in several well-documented properties. PEEK’s elastic modulus has traditionally been cited as 3–4 GPa, overlapping with the upper range reported for cancellous bone (0.1–4 GPa) and theoretically reducing stress shielding and endplate subsidence (Kurtz and Devine, 2007). However, a recent comprehensive review by Horváth et al. (2026) demonstrates that this range is broader than historically appreciated: depending on the manufacturing method—particularly fused deposition modeling (FDM) parameters—PEEK modulus can vary from 2.415 to 8.06 GPa, with Poisson’s ratio of 0.375–0.388 and density of 1.3 g/cm^3^ (versus 4.43 g/cm^3^ for Ti-6Al-4V). For context, human cortical bone has a general modulus of approximately 12–18 GPa (Niinomi, 2008), while cancellous bone ranges from approximately 0.1–4 GPa. It should be noted that cortical bone properties vary by anatomical site; Horváth et al. (2026) report human cranial cortical bone values of 2.032–21 GPa and femoral cortical bone of 9.55–19.9 GPa. Site-specific values for lumbar vertebral bone are not available from existing comparative reviews. PEEK’s radiolucency permits unobstructed postoperative radiographic visualization of fusion progression (Rao et al., 2014), and its chemical inertness and biocompatibility without cytotoxicity or mutagenicity are well established (Rivard et al., 2002).

However, the fundamental limitation of PEEK resides precisely in its bioinert surface chemistry. The hydrophobic aromatic backbone of PEEK resists protein adsorption and cell attachment, failing to promote the cascade of osteoblast adhesion, proliferation, and differentiation that is requisite for direct bone-implant bonding (Sagomonyants et al., 2008). A seminal series of investigations by the Boyan and Schwartz laboratory established the molecular basis of this deficiency. Olivares-Navarrete et al. (2012) demonstrated that osteoblasts cultured on PEEK substrates exhibit a less differentiated phenotype, reduced alkaline phosphatase activity, and diminished bone morphogenetic protein (BMP) production—specifically BMP-2, BMP-4, and BMP-7—compared to those cultured on titanium alloy substrates. Critically, Olivares-Navarrete et al. (2015) subsequently established that PEEK surfaces promote a peri-implant inflammatory milieu characterized by upregulated pro-inflammatory cytokines (IL-1β, IL-6, TNF-α) and fibrogenic factors, whereas microtextured titanium surfaces selectively upregulate osteogenic and angiogenic factors including BMP-2, osteoprotegerin (OPG), and vascular endothelial growth factor (VEGF). Clinically, this biological divergence can manifest as the “PEEK-halo effect”—a radiolucent line surrounding the cage on computed tomography (CT) that represents fibrous tissue encapsulation rather than osseous integration (Phan et al., 2016). The long-term consequence may be a form of failed fusion termed “locked pseudoarthrosis” (Fraser), wherein a PEEK cage appears radiographically stable on static CT imaging but is mechanically isolated from the host bone by a continuous fibrous membrane, ultimately progressing to symptomatic failure requiring revision surgery (Weinberg et al., 2026).

The advent of additive manufacturing (AM)—specifically selective laser melting (SLM) and electron beam melting (EBM)—has inaugurated a new era in interbody cage design by enabling precise, computer-aided control over the three-dimensional architecture of the implant at the macro-, micro-, and nano-scales (Su and Sun, 2026). Through systematic variation of porosity fraction (typically 60%–80%), pore size (300–700 μm), strut topology, and surface texture, 3D-printed porous titanium (3D-pTi) cages can simultaneously achieve: reduction of the effective elastic modulus from the 50–110 GPa of solid Ti-6Al-4V to approximately 2–6 GPa—a range that overlaps with both PEEK and human cancellous bone (Fogel et al., 2022a; Chatham et al., 2017); creation of an interconnected three-dimensional porous network that supports vascular infiltration, osteoprogenitor cell migration, and *de novo* bone formation throughout the implant volume—true bone ingrowth—rather than mere surface apposition (McGilvray et al., 2018); and preservation of the favorable surface chemistry of titanium, which includes a spontaneous titanium dioxide passive layer that promotes osteoblast attachment and hydroxyapatite nucleation (Gittens et al., 2014). The theoretical framework underpinning 3D-pTi design thus represents a transition from the “bioinert spacer” paradigm—in which the cage merely maintains disc height while fusion occurs around it—to a “bioactive scaffold” paradigm—in which the cage actively participates in and accelerates the osteogenic cascade.

The past 4 years have witnessed an unprecedented concentration of high-level comparative evidence. Most notably, Weinberg et al. (2026) reported a single-blinded, prospective randomized controlled trial (RCT) comparing micro- and nano-textured 3D-printed porous titanium cages with PEEK cages in 1- to 2-level transforaminal lumbar interbody fusion (TLIF). The trial was terminated early after a pre-specified interim analysis revealed a statistically significant difference in the primary endpoint: the 6-month CT fusion rate (Brantigan-Steffee-Fraser [BSF] grade 3, defined as continuous trabecular bridging without any radiolucency) was 100% (10/10 levels) for 3D-pTi versus 0% (0/15 levels) for PEEK (P < 0.001). Bayesian mixed-effects logistic regression—employed to overcome the statistical challenge of zero events in the PEEK group—produced a posterior probability exceeding 99.9% that 3D-pTi increased the odds of fusion, with model-predicted 6-month BSF-3 probabilities of 91.2% for 3D-pTi versus 9.0% for PEEK.

Several design features constrain the generalizability of this result. First, the sample size was small (17 patients, 25 interbody levels), precluding subgroup analyses and limiting generalizability; the Bayesian framework quantifies the strength of evidence given the observed data but does not increase clinical precision. Second, the trial excluded patients with osteoporosis, prior lumbar fusion, active infection, and use of medications affecting bone metabolism (corticosteroids, immunosuppressants)—precisely the populations in which the choice of cage material may be most consequential. Third, only two specific cage designs, from single manufacturers, were compared, and the results may not generalize to other 3D-pTi or PEEK designs. Fourth, the 6-month endpoint provides no information on the durability of the fusion interface or long-term clinical outcomes; the BSF-3 threshold is stringent, and it remains unknown whether a PEEK cage achieving BSF-2 at 6 months would progress to BSF-3 by 12 months with equivalent clinical outcomes. Fifth, patient-reported outcome measures improved significantly within the 3D-pTi group at 6 months, but the between-group difference in improvement magnitude was not statistically significant—likely reflecting sample size limitations. These caveats do not invalidate the finding but should constrain the weight it is accorded in clinical decision-making; the trial should not be used alone to justify broad clinical preference for all 3D-pTi cages over all PEEK cages.

Concurrently, four systematic reviews with meta-analyses have been published (Duan et al., 2024; Liu et al., 2024; Pairojboriboon et al., 2024; Wang et al., 2025), alongside large-scale real-world cohort studies (Levy et al., 2026; Jacob et al., 2025; Corso et al., 2025; Burkhard et al., 2025a; Burkhard et al., 2025b), additional RCTs (Toop et al., 2023; Ham et al., 2024), and translational investigations spanning surface science to human retrieval histology (Torstrick et al., 2020; Laratta et al., 2022; van den Brink and Lamerigts, 2020). Despite this evidentiary richness, clear clinical consensus has not emerged. A subset of studies reports that titanium cages paradoxically exhibit higher subsidence rates than PEEK (Hamrick et al., 2026), and regression analyses demonstrate that cage material is not an independent predictor of subsidence after adjustment for geometric confounders such as surface area and shape (Levy et al., 2026). Meanwhile, hybrid strategies—titanium-coated PEEK (TiPEEK), plasma-sprayed PEEK, and additively manufactured porous PEEK—are actively evolving as potential compromises that address PEEK’s biological deficiencies while preserving its mechanical and imaging advantages (Hasegawa et al., 2020; Torstrick et al., 2018).

Prior reviews have covered elements of the mechanistic background for 3D-pTi cages. Toop et al. (2021) reviewed activated titanium cage design and proposed frameworks for clinical trial design, while Muthiah et al. (2022) comprehensively traced the evolution of PEEK and titanium interbody devices, incorporating surface technologies, material combinations, and early clinical results through 2021. The present review builds upon these contributions and makes three incremental advances: (a) Cross-scale integration—prior reviews each covered segments of the evidence chain in isolation, whereas this review uniquely connects *in vitro* surface science → the Torstrick topography-versus-chemistry experiment → large-animal interbody models → human retrieval histology → RCTs → meta-analyses into a continuous translational argument. (b) The “material versus design” framework—the field has often conflated material identity (titanium vs. PEEK) with structural design (solid vs. porous vs. coated); this review explicitly disentangles these two dimensions. (c) Post-2022 evidence synthesis—the Weinberg RCT (2026), Ham RCT (2024), four meta-analyses (Duan et al., 2024; Liu et al., 2024; Pairojboriboon et al., 2024; Wang et al., 2025), and large-scale cohort studies (Levy et al., 2026; Jacob et al., 2025; Burkhard et al., 2025a; Burkhard et al., 2025b) were all published after the literature cutoffs of earlier reviews and substantially change the evidentiary landscape.

This review was designed to address three overarching questions: (1) What is the mechanistic basis—spanning surface science, biomechanics, and bone biology—by which 3D-pTi cages may accelerate fusion relative to PEEK? (2) Across the evidence hierarchy, how consistent is the clinical evidence for accelerated fusion, reduced subsidence, and lower revision rates, and what are the critical interpretive limitations? (3) Under which specific clinical scenarios does the evidence support a preferential role for 3D-pTi, and where does PEEK (or TiPEEK) remain a rational and well-supported choice?

## 2 Search strategy and selection criteria

This review employed a structured, comprehensive literature search combined with narrative synthesis. Reporting was guided by the SANRA (Scale for the Assessment of Narrative Review Articles) guidelines (Baethge et al., 2019). Consistent with the narrative nature of this synthesis, formal quality assessment of individual studies and quantitative pooling were not performed; accordingly, this review does not claim to be a systematic review.

### 2.1 Search strategy

A structured search was conducted in PubMed, Web of Science, EMBASE, and the Cochrane Library from database inception to May 2026. The search comprised three complementary strategies designed to capture both direct comparative and mechanistic evidence:

#### 2.1.1 Strategy A (primary—head-to-head comparisons)

Targeted studies directly comparing 3D-printed porous titanium and PEEK cages in lumbar fusion by combining four concept groups using Title/Abstract fields: (1) additive manufacturing terminology (“3D print*”, “three-dimensional print*”, “additive manufactur*”, “porous titanium”, “3D-printed titanium”), (2) material descriptors (“polyetheretherketone”, “PEEK”), (3) procedure terms (“lumbar fusion”, “lumbar interbody fusion”, “TLIF”, “PLIF”, “ALIF”, “LLIF”, “spinal fusion”, “spine surgery”), and (4) implant terminology (“cage”, “interbody cage”, “intervertebral cage”).

#### 2.1.2 Strategy B (supplementary 1 — broad cage material comparisons)

Captured studies comparing cage materials without explicit material terminology in titles or abstracts, using terms such as “cage material” or “interbody material” combined with procedure terms and comparison operators (“comparison”, “versus”, “vs”).

#### 2.1.3 Strategy C (supplementary 2 — mechanistic osseointegration studies)

Captured studies on 3D-pTi osseointegration in spinal applications that did not include PEEK as a comparator, combining additive manufacturing terms with osseointegration terminology (“osseointegration”, “osteointegration”, “bone ingrowth”) and spinal context terms (“spine”, “lumbar”) with methodologic filters (“*in vitro*”, “histolog*”, “cell”).

The complete search strings for all three strategies in all four databases are provided in Supplementary File S1. Additional references—including foundational studies on PEEK biology (Olivares-Navarrete et al., 2012; 2015; Kurtz and Devine, 2007), classic biomechanics (Polikeit et al., 2003), prior reviews (Muthiah et al., 2022; Toop et al., 2021; Enache et al., 2026), and finite element analyses (Carpenter et al., 2018; Zhang et al., 2018)—were identified through citation tracking of included studies and expert knowledge of the field.

### 2.2 Eligibility criteria

Studies were eligible for inclusion if they: (1) directly compared 3D-printed porous titanium, activated titanium, or titanium-coated PEEK cages with unmodified PEEK cages in lumbar interbody fusion (for clinical, radiographic, or biomechanical endpoints); or (2) provided mechanistic data on the osseointegration or biomechanical performance of porous titanium structures relevant to lumbar interbody applications. Eligible study designs included randomized controlled trials, prospective and retrospective cohort studies, systematic reviews and meta-analyses, large-animal interbody fusion models, human retrieval histology, and *in vitro* surface science investigations.

Studies were excluded if they: (1) involved cervical or thoracic fusion exclusively, without a lumbar subgroup; (2) used solid (non-porous, non-activated) titanium as the sole titanium comparator without a distinct porous or surface-modified titanium group; (3) were conference abstracts without full-text availability; or (4) were published in languages other than English.

### 2.3 Study selection and data extraction

Records retrieved from the four databases were deduplicated and screened by title and abstract by two independent reviewers; disagreements were resolved through discussion. Full texts of potentially eligible reports were retrieved and assessed against the eligibility criteria by the two reviewers. Consistent with the narrative review format, no PRISMA flow diagram or study-selection count is presented; the reference list represents the complete set of studies synthesized in this review. Data pertaining to study design, cage characteristics, and outcome measures were extracted by the authors.

### 2.4 Data synthesis

Given the substantial heterogeneity in study design, cage architecture (porosity, pore size, lattice topology), surgical technique, graft materials, and outcome definitions across the included literature, a narrative qualitative synthesis was performed rather than quantitative meta-analysis. Studies were grouped thematically by evidence domain—surface science (*in vitro*), animal interbody fusion models, human retrieval histology, clinical comparative evidence (RCTs, cohort studies, and meta-analyses), and health economic considerations—and synthesized within the analytical framework that distinguishes between material identity and implant design as independent determinants of clinical performance.

## 3 Material properties and the bone-implant interface

### 3.1 PEEK: physical properties and biological limitations

PEEK is a semi-crystalline thermoplastic belonging to the polyaryletherketone family. Its mechanical properties have been extensively characterized. The Young’s modulus of PEEK—traditionally cited as 3–4 GPa—has been shown by Horváth et al. (2026) to span a broader range of 2.415–8.06 GPa depending on manufacturing parameters, particularly FDM processing conditions, with Poisson’s ratio of 0.375–0.388 and density of 1.3 g/cm^3^ (versus 4.43 g/cm^3^ for Ti-6Al-4V). This places PEEK within the range of human cancellous bone (0.1–4 GPa) at its lower values and approaching cortical bone only at its upper processing-dependent extreme. For context, general human cortical bone has a modulus of approximately 12–18 GPa (Niinomi, 2008). Cortical bone properties vary considerably by anatomical site: Horváth et al. (2026) report cranial cortical bone values of 2.032–21 GPa and femoral cortical bone of 9.55–19.9 GPa. Site-specific values for lumbar vertebral bone, which would be most relevant to the present review, are not available from existing comparative reviews. PEEK’s radiolucency, chemical stability, and well-established manufacturing protocols have made it the default choice for interbody cages for over two decades (Kurtz and Devine, 2007). The clinical relevance of elastic modulus matching in interbody implant selection was reviewed by Heary et al. (2017), who emphasized that an ideal cage should approximate the modulus of the vertebral body it replaces to minimize stress shielding while maintaining sufficient stiffness to resist subsidence.

The biological limitation of PEEK is not its bulk mechanical properties but its surface chemistry. The aromatic backbone, while conferring chemical stability, presents a hydrophobic surface that resists protein adsorption—the initial step required for integrin-mediated cell attachment (Sagomonyants et al., 2008). Without an adsorbed protein layer, osteoblast precursor cells fail to adhere, spread, and initiate the differentiation cascade. Olivares-Navarrete et al. (2012) provided direct comparative data: human osteoblasts cultured on PEEK exhibited lower alkaline phosphatase activity and produced significantly less BMP-2, BMP-4, and BMP-7 than those cultured on Ti-6Al-4V. This was not merely a quantitative difference but a qualitative shift in cellular phenotype—osteoblasts on PEEK were less differentiated and less capable of driving matrix mineralization. The 2015 study from the same group (Olivares-Navarrete et al., 2015) demonstrated that the peri-implant inflammatory environment differs fundamentally between materials. PEEK surfaces promoted a pro-inflammatory, pro-fibrotic milieu (IL-1β↑, IL-6↑, TNF-α↑), whereas microtextured titanium surfaces promoted an osteogenic and angiogenic milieu (BMP-2↑, OPG↑, VEGF↑). The clinical translation of this molecular divergence is the “PEEK-halo effect”—a radiolucent line visible on CT that represents the fibrous membrane interposed between the PEEK surface and host bone (Phan et al., 2016).

### 3.2 3D-pTi: design logic and structural characteristics

3D-pTi cages are produced by SLM or EBM of Ti-6Al-4V powder, building the implant layer by layer from a computer-aided design file. The key design variables—porosity fraction (typically 60%–80%), pore size (300–700 μm), strut thickness, unit cell topology, and surface texture—can be independently controlled, enabling systematic optimization of mechanical and biological performance (Su and Sun, 2026). Taniguchi et al. (2016) established in a rabbit tibia model that pore sizes of 500–700 μm produce maximal bone ingrowth and vascularization; this range is precisely achievable by current AM technologies.

The reduction in effective elastic modulus from approximately 50–110 GPa (solid Ti-6Al-4V) to approximately 2–6 GPa (porous structure) is achieved through the removal of material volume in a controlled lattice architecture, not through alloy modification (Fogel et al., 2022a; Chatham et al., 2017). Critically, the interconnected porosity creates a three-dimensional scaffold that supports vascular infiltration, osteoprogenitor cell migration, and *de novo* bone formation throughout the implant volume—a mode of biological fixation termed “bone ingrowth” or “in-growth”—in contrast to the two-dimensional bone apposition (“on-growth”) achievable on solid or coated surfaces. Tsai et al. (2016) demonstrated that biomechanical performance varies significantly with structural design parameters in porous AM cages, and Yee-Yanagishita et al. (2022) showed that porous titanium cages with optimized body lattice and microporous endplates achieved the highest subsidence resistance compared with porous PEEK and truss titanium designs. At the cellular level, laser-sintered constructs with bio-inspired porosity and surface micro/nano-roughness have been shown to enhance mesenchymal stem cell differentiation and matrix mineralization *in vitro* (Cheng et al., 2016), providing a mechanistic basis for the osteoconductive properties of additively manufactured titanium observed *in vivo*.

It is essential to distinguish among the several categories of titanium-based cage technologies that appear in the comparative literature, as they differ fundamentally in structural architecture, biological mechanism, and clinical evidence base:

- Activated titanium cages feature nano-etched surface texture and micro-lattice architecture but do not possess an interconnected three-dimensional porous network throughout the implant volume. Their biological mechanism is two-dimensional bone apposition (on-growth), with enhanced osteoblast differentiation driven by surface topography at the nano- and microscale. Representative clinical evidence includes the randomized controlled trial by Toop et al. (2023), which compared activated titanium cages with PEEK in 1- and 2-level TLIF.
- 3D-printed porous titanium (3D-pTi) cages are characterized by an interconnected, fully porous architecture throughout the implant volume. Typical design parameters include 60%–80% porosity and pore sizes ranging from 300 to 700 μm. Their biological mechanism is three-dimensional bone ingrowth (in-growth), with vascular infiltration and *de novo* bone formation occurring throughout the implant rather than solely at the implant–endplate interface. Representative clinical evidence includes the randomized trial by Weinberg et al. (2026), the multicenter RCT by Ham et al. (2024), and multiple non-randomized comparative cohort studies.
- Titanium-coated PEEK (TiPEEK) cages consist of a two-dimensional titanium or hydroxyapatite coating applied to a solid PEEK core. Their biological mechanism is surface bone apposition only; the solid PEEK core precludes three-dimensional bone ingrowth through the implant volume. Representative clinical evidence includes the multicenter randomized trial by Hasegawa et al. (2020) and the systematic review and meta-analysis by Mariscal et al. (2025).
- Solid titanium cages are machined from solid Ti-6Al-4V without interconnected porosity. They offer osteoconductive titanium surface chemistry but lack the porous architecture required for bone ingrowth. Earlier meta-analyses comparing solid titanium with PEEK yielded heterogeneous and inconclusive results (Seaman et al., 2017; Tan et al., 2021), underscoring that titanium surface chemistry alone—in the absence of a porous architecture—may not confer a consistent fusion advantage.

Clinical outcomes reported for one category should not be indiscriminately extrapolated to another. This distinction is central to the thesis of the present review: under the experimental and clinical conditions studied to date, the porous architecture—rather than the elemental identity of titanium per se—appears to be the predominant driver of osseointegration. However, this principle has not been universally validated across all cage designs, surgical techniques, and patient populations.

### 3.3 Surface topography as the dominant variable: the torstrick experiment

A foundational question in implant osseointegration research has been whether surface chemistry (material identity) or surface topography (physical architecture) exerts the greater influence on bone-implant fixation. Torstrick et al. (2020) conducted an experiment that provided quantitative data addressing this question under specific experimental conditions. Using PEEK substrates, they fabricated three distinct surface topographies—smooth, roughened (grit-blasted), and porous—and then applied an ultrathin (∼30 nm) titanium dioxide (TiO_2_) coating via atomic layer deposition (ALD) to independently manipulate surface chemistry without altering topography. Cylindrical implants representing all six combinations of topography × chemistry (PEEK vs. TiO_2_) were implanted into the proximal tibial metaphyses of rats and evaluated at 8 weeks by micro-computed tomography (μCT), histology, and biomechanical pull-out testing.

Under the specific experimental conditions tested—a rat tibial implant model using implants with approximately 72 μm pore size and an 8-week observation endpoint—Torstrick et al. (2020) demonstrated that surface topography accounted for approximately 65% of the variance in implant fixation strength versus approximately 6% for surface chemistry. This result suggests that topography is the predominant factor governing osseointegration in this model. Porous surfaces generated significantly greater fixation strength than roughened or smooth surfaces irrespective of surface chemistry (P < 0.05). The bone ingrowth fraction was 38.9% ± 2.8% in porous PEEK versus 30.7% ± 3.3% in porous TiO_2_ (P = 0.07). The direction of the effect (topography > chemistry) is broadly consistent with findings from large-animal interbody fusion models (Fogel et al., 2022b; McGilvray et al., 2018) and earlier surface-science investigations (Gittens et al., 2014). However, the precise variance partitioning should be understood as model-specific rather than universally generalizable. Whether this relative weighting extrapolates to the mechanically loaded, highly vascular human lumbar interbody environment—where loading magnitudes, endplate bone density, and healing biology differ substantially from a rat tibial defect—remains to be established. The pore size in this experiment (∼72 μm) is substantially smaller than the 300–700 μm range used in clinical 3D-pTi cages, representing an additional caveat for direct clinical translation.

This principle has been corroborated by earlier work from the same group demonstrating that porous PEEK significantly enhances *in vitro* osteoblast proliferation and cell-mediated mineralization compared to smooth PEEK (Torstrick et al., 2016) and that the porous architecture outperforms plasma-sprayed titanium-coated PEEK in bone-implant contact and interfacial stiffness (Torstrick et al., 2018). Gittens et al. (2014) provided complementary evidence that implant surface microroughness and nanostructures promote osteoblast differentiation—characterized by elevated alkaline phosphatase, osteopontin, and BMP expression—while smooth, polished surfaces are bio-inert. Collectively, these data establish that the biological “inertness” historically attributed to PEEK is, to a substantial degree, a topographical rather than a chemical phenomenon, and that the creation of interconnected porosity is the single most impactful design variable for achieving osseointegration demonstrated to date.

The mechanistic framework linking surface topography to clinical outcomes is summarized in Figure 1.

**FIGURE 1:**
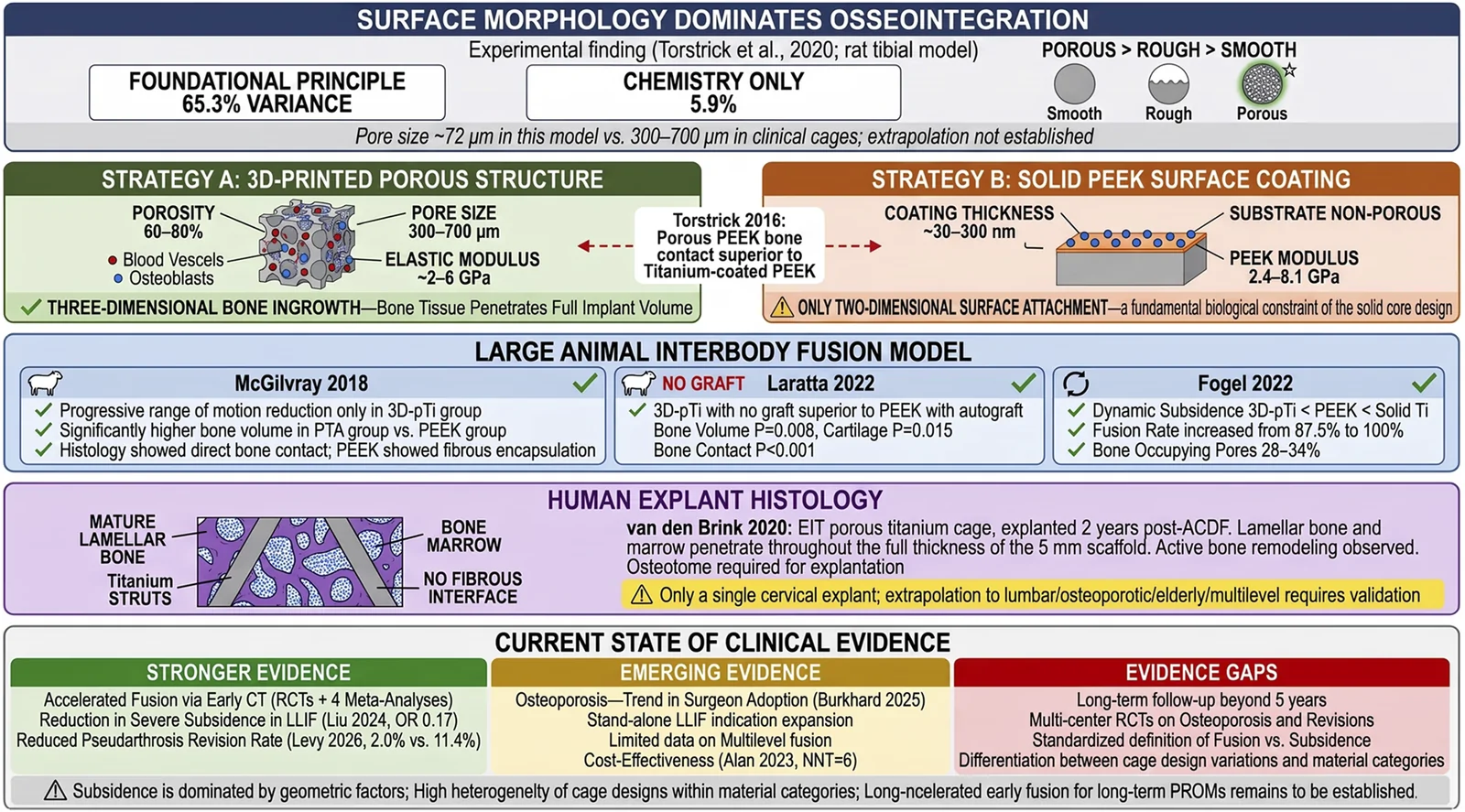
Mechanistic framework linking surface topography to clinical outcomes for 3D-printed porous titanium versus PEEK interbody cages. (Layer 1) Experimental finding: in a rat tibial model, surface topography accounted for approximately 65% of variance in osseointegration versus approximately 6% for chemistry (Torstrick et al., 2020); the precise variance partitioning is model-specific, and extrapolation to the human lumbar interbody environment has not been established (pore size in this model ∼72 μm vs. 300–700 μm in clinical cages). (Layer 2) Two design strategies: 3D-printed porous architecture enables bone ingrowth throughout the implant volume, whereas surface coatings on solid PEEK enable only two-dimensional bone apposition—a fundamental biological constraint of the solid core design (Torstrick et al., 2018). (Layer 3) Large-animal validation: 3D-pTi cages without bone graft outperform PEEK cages with autograft (Laratta et al., 2022); only 3D-pTi shows progressive biomechanical stabilization (McGilvray et al., 2018); dynamic subsidence is lowest with 3D-pTi (Fogel et al., 2022). (Layer 4) Human retrieval histology: mature lamellar bone in direct contact with titanium scaffold throughout the full 5-mm height, with active remodeling and no fibrous interposition (van den Brink and Lamerigts, 2020). (Layer 5) Clinical evidence stratified by strength (stronger, emerging, gaps), with key studies cited. The bottom caution emphasizes that subsidence is geometry-dependent, cage designs within material classes are heterogeneous, and the clinical significance of accelerated early fusion for long-term patient-reported outcomes remains to be established. Created with BioRender.

### 3.4 Large-animal interbody fusion models

The rodent long-bone model employed by Torstrick et al. provides high-throughput, well-controlled mechanistic data but does not replicate the complex loading conditions, limited vascular supply, and constrained bone-healing environment of the human interbody space. McGilvray et al. (2018) directly compared three cage types—PEEK, plasma-sprayed porous titanium-coated PEEK (PSP), and 3D-printed porous titanium alloy (PTA; Tritanium®)—in a skeletally mature ovine lumbar interbody fusion model (L2–L3 and L4–L5, lateral retroperitoneal approach) with autologous iliac crest bone graft (AICBG), sacrificing animals at 8 and 16 weeks. The principal findings were as follows: (i) at 16 weeks, range of motion (ROM) in flexion-extension was significantly lower in the PTA group compared to PEEK (P = 0.02); (ii) only the PTA group demonstrated a statistically significant reduction in ROM and increase in stiffness across all three loading directions (flexion-extension, lateral bending, axial rotation) between the 8- and 16-week time points (P ≤ 0.01), indicating progressive biological stabilization unique to the porous titanium architecture; (iii) μCT analysis revealed significantly greater total bone volume within the graft window for PTA cages compared to PEEK at both 8 and 16 weeks (P < 0.01); and (iv) histologically, PEEK cages were surrounded by fibrous connective tissue, whereas PTA cages exhibited new bone and vascular infiltration throughout the porous structure, with direct bone-to-implant contact and no interposed fibrous layer.

Laratta et al. (2022) employed a deliberately asymmetric comparison designed to test a provocative hypothesis: can a 3D-pTi cage without any bone graft outperform a PEEK cage supplemented with AICBG—the historical gold standard for spinal fusion? In an ovine lumbar interbody fusion model, 3D-pTi cages (TRUST™) implanted empty were compared to PEEK cages packed with AICBG at 4 and 8 weeks postoperatively. The 3D-pTi group demonstrated significantly greater bone volume fraction (P = 0.008), cartilaginous area (P = 0.015), and bone-implant contact (BIC; P < 0.001) at both time points. Histological examination revealed that PEEK cages lacked osseointegration, with fibrous tissue concentrated at the bone-implant interface and no new bone formation within the graft window. In contrast, 3D-pTi cages exhibited direct integration with host bone, with woven and lamellar bone distributed along the cage struts, and the simultaneous presence of cartilage and collagen in the central cage region—indicating activation of both endochondral and intramembranous ossification pathways.

These findings received convergent validation from several independent laboratories. Van Horn et al. (2021) confirmed that 3D-printed titanium alloy cages achieve “complete bony ingrowth and ongrowth” histologically superior to both standard titanium alloy and PEEK cages in the ovine model. Guyer et al. (2016) demonstrated in a porcine model that the interface shear strength of deeply porous titanium scaffolds (10.2 MPa) was significantly greater than that of PEEK (1.5 MPa) and allograft (3.1 MPa). McGilvray et al. (2017) demonstrated that a PEEK/titanium composite interbody spacer improved the bone-implant interface compared to pure PEEK in the ovine LLIF model.

Fogel et al. (2022b) performed a comprehensive mechanical–biological characterization of 3D-pTi cages with stress-optimized body lattice and microporous endplates (Modulus XLIF®) that offers the most instructive quantitative data to date. In static axial compression testing (ASTM F2077), 3D-pTi cage stiffness was intermediate between solid PEEK (lowest) and solid titanium (highest). In static subsidence testing (ASTM F2267), however, 3D-pTi block stiffness was comparable to solid titanium and significantly greater than solid PEEK—indicating that 3D-pTi combines lower structural stiffness with higher local resistance to endplate penetration. Most clinically relevant, in dynamic subsidence testing under cyclic loading simulating activities of daily living, 3D-pTi demonstrated the smallest subsidence displacement among all tested materials. At 12 and 26 weeks post-implantation in the ovine model, the 3D-pTi cage achieved fusion rates of 87.5% and 100%, respectively, with bone occupying 28%–34% of the available pore volume.

Finite element analyses (FEA) provide a complementary computational perspective. Zhang et al. (2018) modeled various porous AM cage configurations in TLIF and demonstrated that, relative to solid designs, porous architectures simultaneously reduced endplate stress and intra-cage stress, promoting more homogeneous load distribution. Carpenter et al. (2018) conducted an FEA that introduced bone ingrowth as a time-dependent variable, comparing porous PEEK and porous titanium. At simulated 4-week ingrowth, porous PEEK shared a significantly larger proportion of the total load with the ingrown bone (∼66%) compared to porous titanium (∼13%), and generated higher physiological strains in adjacent bone. This finding should not be misinterpreted as evidence that PEEK is mechanically superior; rather, it illustrates that the optimal design may involve intermediate effective stiffness—sufficient to share load with bone (avoiding stress shielding) but not so compliant as to permit excessive micromotion. The design space for 3D-pTi—modulating porosity, pore size, strut thickness, and lattice topology—provides precisely this capacity for fine-tuning.

### 3.5 Human retrieval histology

The most direct method for validating osseointegration is histological analysis of human explanted implants. van den Brink and Lamerigts (2020) reported the analysis of an EIT Cellular Titanium® cervical cage retrieved 2 years after C5/C6 anterior cervical discectomy and fusion (ACDF) without an anterior plate, at the time of revision surgery for adjacent-level C6/C7 degeneration. Preoperative radiographs confirmed complete radiological integration of the cage at both superior and inferior endplates. Intraoperatively, the surgeon required a chisel to dislodge the cage—an observation that attests to the mechanical tenacity of the bone-implant interface.

Macroscopically, white tissue indicative of bone covered both the superior and inferior surfaces of the explanted cage. Histological examination using Masson Goldner trichrome staining demonstrated mature lamellar bone (stained light blue) with interspersed bone marrow throughout the entire 5-mm height of the porous titanium scaffold. Young woven bone and osteoid (stained red) were also visible, indicating ongoing physiological remodeling. Critically, trabecular bone was observed in direct contact with the titanium struts—without any fibrous tissue interface—and bone was present even in the central portion of the cage. This latter observation has important biomechanical implications: the presence of viable bone in the cage center suggests that physiological loading is transmitted through the full thickness of the implant, consistent with Wolff’s law governing load-dependent bone maintenance, and argues against clinically significant stress shielding within the porous scaffold.

Although published human retrieval histology of 3D-pTi cages remains limited to case reports and small series—a critical evidence gap—the concordance of these findings with the animal data described above (McGilvray et al., 2018; Laratta et al., 2022; Van Horn et al., 2021) constitutes a coherent translational chain from *in vitro* → small animal → large animal → human.

### 3.6 From bench to bedside: a cautionary note

The translational chain described above—in vitro → small animal → large animal → human retrieval—provides compelling biological plausibility for the porous architecture advantage. These mechanistic data establish that interconnected porosity creates a fundamentally different mode of biological fixation than that achievable with solid or coated implants. However, these data should not be overextended into firm clinical recommendations without acknowledging the translational gap. Animal interbody fusion is performed in young, healthy animals with controlled surgical conditions and standardized defect sizes, whereas human lumbar fusion is typically performed in older patients with variable bone quality, medical comorbidities (diabetes, smoking, osteoporosis), and greater surgical complexity (multilevel constructs, revision surgery). The clinical evidence is examined in Sections 4–6, and the gap between mechanistic promise and clinical proof is explicitly addressed throughout.

The preclinical and biomechanical evidence is summarized in Table 1.

**TABLE 1:** Summary of preclinical and biomechanical studies informing the biological rationale for 3D-printed porous titanium interbody cages.

Study	Model/Design	Comparison	Key endpoints	Principal finding
Olivares-Navarrete et al. (2012)	*In vitro* (human osteoblasts)	Ti-6Al-4V vs PEEK	Osteoblast differentiation, BMP production	Ti alloy surfaces promoted more differentiated osteoblast phenotype and significantly higher BMP-2, BMP-4, and BMP-7 production vs PEEK
Olivares-Navarrete et al. (2015)	*In vitro* (macrophages, lymphocytes)	Microtextured Ti vs PEEK	Peri-implant inflammatory cytokine profile	PEEK promoted pro-inflammatory (IL-1β, IL-6, TNF-α) and fibrogenic factors; microtextured Ti upregulated osteogenic (BMP-2, OPG) and angiogenic (VEGF) factors
Torstrick et al. (2016)	*In vitro* + rat tibia	Porous vs smooth PEEK	Porosity effect on mechanics and cell response	Porous PEEK enhanced osteoblast proliferation and cell-mediated mineralization vs smooth PEEK
Taniguchi et al. (2016)	*In vivo* (rabbit tibia)	Porous Ti (300/600/900 μm pore)	Optimal pore size for bone ingrowth	500–700 μm pores provided maximal bone ingrowth and vascularization; this range is precisely achievable by SLM/EBM
Guyer et al. (2016)	*In vivo* (porcine)	Porous Ti scaffold vs PEEK vs allograft	Interface shear strength	Porous Ti (10.2 MPa) >> PEEK (1.5 MPa); allograft (3.1 MPa); P < 0.05
McGilvray et al. (2017)	*In vivo* (ovine LLIF)	PEEK/Ti composite vs PEEK	ROM, μCT bone volume, histology	Ti/PEEK composite improved bone-implant interface vs pure PEEK; trend toward improved fusion
McGilvray et al. (2018)	*In vivo* (ovine LLIF, 8 and 16 wk)	3D-pTi (PTA) vs PSP vs PEEK	ROM reduction, μCT bone volume, histology	Only PTA group showed significant ROM reduction 8→16 wk (P ≤ 0.01); bone volume significantly higher in PTA vs PEEK at both time points (P < 0.01); direct bone-implant contact in PTA, fibrous encapsulation in PEEK
Torstrick et al. (2018)	*In vivo* (rat tibia)	Porous PEEK vs Ti-coated PEEK	Bone-implant contact, fixation	Porous PEEK significantly outperformed Ti-coated PEEK in bone-implant contact; architecture > coating
Carpenter et al. (2018)	FEA (simulated bone ingrowth)	Porous Ti vs porous PEEK	Load sharing with ingrown bone	At 4-wk ingrowth, porous PEEK shared ∼66% load vs porous Ti ∼13%; higher physiological strain in adjacent bone with PEEK
Zhang et al. (2018)	FEA (TLIF model)	Porous AM vs solid cages	Endplate stress, intra-cage stress	Porous AM cages simultaneously reduced endplate stress and intra-cage stress, promoting homogeneous load distribution
Torstrick et al. (2020)	*In vivo* (rat tibia)	6 combinations: 3 topographies × 2 chemistries (PEEK/TiO_2_ via ALD)	Fixation strength (pull-out), ANOVA variance partitioning	Topography explained ∼65% variance (ω^2^ ≈ 0.65); chemistry ∼6% (ω^2^ ≈ 0.06); porous surfaces superior regardless of chemistry (P < 0.05); bone ingrowth: porous PEEK 38.9% vs porous TiO_2_ 30.7% (P = 0.07). Note: rat tibial model; pore size ∼72 μm; extrapolation to human lumbar interbody environment not established
Van Horn et al. (2021)	*In vivo* (ovine)	3D-pTi vs solid Ti vs PEEK	Histology, bone ingrowth/ongrowth	3D-pTi achieved “complete bony ingrowth and ongrowth”; superior to both solid Ti and PEEK
Laratta et al. (2022)	*In vivo* (ovine lumbar, 4 and 8 wk)	3D-pTi (no graft) vs PEEK + AICBG	Bone volume fraction, cartilage area, BIC	3D-pTi (no graft) outperformed PEEK + AICBG on all metrics: bone volume P = 0.008, cartilage P = 0.015, BIC P < 0.001; both endochondral and intramembranous ossification activated
Fogel et al. (2022a)	*In vitro* + *in vivo* (ovine, 12 and 26 wk)	3D-pTi (Modulus XLIF) vs solid PEEK vs solid Ti	Static/dynamic mechanical testing; *in vivo* fusion	Dynamic subsidence: 3D-pTi < solid PEEK < solid Ti; *in vivo* fusion: 87.5% (12 wk), 100% (26 wk); bone occupying 28%–34% pore volume
van den Brink and Lamerigts (2020)	Human retrieval (cervical, 2 yr post-ACDF)	EIT Cellular Titanium cage explant histology	Histomorphology (Masson Goldner)	Mature lamellar bone + marrow throughout 5 mm scaffold height; direct bone-Ti contact without fibrous interposition; active remodeling (blue lamellar + red woven bone/osteoid)

Abbreviations: AICBG, autologous iliac crest bone graft; ALD, atomic layer deposition; AM, additive manufacturing; BIC, bone-implant contact; BMP, bone morphogenetic protein; EBM, electron beam melting; FEA, finite element analysis; PTA, 3D-printed porous titanium alloy; PSP, plasma-sprayed porous titanium-coated PEEK; ROM, range of motion; SLM, selective laser melting.

## 4 Does 3D-pTi accelerate early fusion?

### 4.1 Randomized controlled trials: 3D-pTi versus PEEK

The single-blinded, prospective RCT by Weinberg et al. (2026) at the Ohio State University Wexner Medical Center is the most discussed comparative study in this field. The trial randomized patients undergoing 1–2 level TLIF to receive either a micro- and nano-textured 3D-pTi cage (Adaptix™, n = 10 levels) or a PEEK cage (Capstone™, n = 15 levels), with the primary endpoint being 6-month CT-assessed fusion according to the BSF grading system. The trial was terminated early after a pre-specified interim analysis revealed a statistically significant difference in the primary endpoint: the 3D-pTi group demonstrated a BSF-3 fusion rate of 100% (10/10), whereas the PEEK group demonstrated 0% (0/15) (P < 0.001).

The complete separation of the data—zero events in one arm—rendered standard frequentist logistic regression unreliable. The authors therefore employed Bayesian mixed-effects logistic regression with weakly informative priors. The posterior probability that 3D-pTi increased the odds of achieving BSF-3 fusion exceeded 99.9%, corresponding to a near-certain beneficial effect. Model-predicted 6-month BSF-3 fusion probabilities were 91.2% for 3D-pTi versus 9.0% for PEEK. The two groups did not differ significantly in age, BMI, number of fused levels, cage height, length of hospital stay, operative duration, or perioperative complications (including subsidence and reoperation events).

The interpretive caveats enumerated in the Introduction apply. Several design features constrain the generalizability of this result: (1) the sample size was small (17 patients, 25 interbody levels); (2) the trial excluded patients with osteoporosis, prior lumbar fusion, and medications affecting bone metabolism—populations in which cage material selection may be most consequential; (3) only two specific cage designs, from single manufacturers, were compared; and (4) the 6-month endpoint provides no information on the durability of the fusion interface or long-term clinical outcomes. While this trial provides confirmatory randomized evidence for accelerated early radiographic fusion with 3D-pTi—a finding directionally consistent with that reported by Toop et al. (2023) using a different titanium cage technology (activated titanium with nano-etched surface)—these constraints preclude its use as standalone justification for broad clinical preference. The trial’s principal contribution is the demonstration, in a randomized design, of the biological principle that a porous titanium architecture can achieve rapid, reliable osseous integration in the human lumbar interbody environment.

Toop et al. (2023) provided convergent RCT evidence with a larger sample. This study compared activated titanium cages—featuring nano-etched surface texture and a micro-lattice architecture that recapitulates key topographical features of 3D-pTi but does not constitute an interconnected three-dimensional porous architecture—with PEEK cages in 1–2 level TLIF. At 6 months, the activated titanium group achieved BSF-3 fusion in 84.0% of levels versus 20.6% in the PEEK group (P < 0.001). Subsidence rates were 20.8% versus 41.4%, respectively (P < 0.001), and the severity of subsidence was also significantly lower in the activated titanium arm. The larger sample in this trial and the consistent direction and magnitude of the treatment effect across two independently conducted RCTs substantially strengthen the evidence base for the hypothesis that surface topography accelerates fusion, though it should be noted that activated titanium and 3D-pTi represent distinct categories of the titanium-based cage family (see Section 3.2).

### 4.2 Design-internal evidence: porous architecture adequacy without a graft chamber

Ham et al. (2024) addressed a complementary question in their multicenter RCT that does not compare titanium against PEEK but rather examines whether the porous titanium architecture alone—without a graft chamber—is sufficient for fusion. Patients undergoing PLIF were randomized to receive either a non-window-type (NW-type) 3D-pTi cage (no graft cavity; fusion is entirely dependent on bone ingrowth through the porous walls) or a window-type (W-type) 3D-pTi cage (containing a central graft chamber). Both arms used 3D-printed porous titanium cages. At 12 months, the NW-type cage achieved a fusion rate of 96.6% versus 93.8% in the W-type group (difference not statistically significant). Notably, the intra-cage osseointegration score (SOR) was significantly higher in the NW-type group (P = 0.007), and the trabecular bone remodeling (TBR) positivity rate was numerically higher (89.7% vs. 78.1%). These results provide clinical proof-of-concept for the finding from the Laratta et al. ovine experiment—that the porous titanium surface alone can support successful interbody fusion without supplemental bone graft—and represent a significant simplification of the surgical workflow.

It should be explicitly noted that this study provides design-internal evidence (non-window-type vs. window-type 3D-pTi) and does not constitute a 3D-pTi versus PEEK comparison. Its relevance to the present review lies in its demonstration that the porous titanium architecture alone—without a central graft chamber or supplemental bone graft—is sufficient to support successful interbody fusion, providing clinical proof-of-concept for the biological principle identified in the ovine experiment by Laratta et al. (2022).

### 4.3 Meta-analytic synthesis

Four independent systematic reviews with meta-analyses have been published as of mid-2026, and their convergent conclusions reinforce the individual-study findings.

Duan et al. (2024) included nine comparative studies and reported that the fusion advantage of 3D-pTi was most pronounced at the 6-month time point, with the difference narrowing but persisting to 12 months. The 1-year subsidence rate was significantly lower in the 3D-pTi group, while complication and reoperation rates showed a favorable trend without reaching statistical significance.

Liu et al. (2024) performed a focused meta-analysis of subsidence in LLIF, including 3 studies (265 patients, 441 levels). The overall subsidence odds ratio (OR) for 3D-pTi versus PEEK was 0.25 (95% CI 0.14–0.44, P < 0.00001). The reduction in severe (Marchi grade III) subsidence was even more pronounced, with an OR of 0.17 (95% CI 0.07–0.46, P = 0.0004). This finding is of direct clinical relevance for LLIF, a procedure in which subsidence directly undermines the indirect decompression that is the procedure’s principal advantage.

Pairojboriboon et al. (2024) performed the only network meta-analysis (NMA) published to date, including 13 TLIF studies (1,919 patients, 1,981 levels) comparing seven cage materials. Although no pairwise comparisons reached statistical significance—a common limitation of NMA when individual studies are small and heterogeneous—the surface under the cumulative ranking curve (SUCRA) provided a clear probability-based ranking: 3D-pTi 0.8 (highest) > titanium-coated PEEK (TiPEEK) 0.6 > solid titanium 0.5 ≈ allograft 0.5 > autograft 0.4 ≈ CFRP 0.4 > PEEK 0.3 (lowest). This ranking is consistent with the framework that the three highest-ranked materials all feature either porous architecture (3D-pTi) or surface-modified titanium (TiPEEK, solid Ti). SUCRA values represent the cumulative probability that a given treatment occupies each possible rank in a simulated sampling distribution, and should be interpreted as treatment-ranking probabilities rather than as a directly causal biological hierarchy or a quantitative dose–response gradient. The clinical implication is that while the evidence consistently suggests a ranking of fusion performance, the magnitude and certainty of the differences between adjacent ranks—particularly between 3D-pTi and TiPEEK—remain imprecisely estimated.

Wang et al. (2025) conducted the largest pairwise meta-analysis (19 studies, 2,057 patients) and performed the critical subgroup analysis distinguishing 3D-printed titanium from non-3D (solid) titanium. The results revealed a clear “subgroup effect”: in the 3D-Ti subgroup, fusion rates were significantly higher, and subsidence and reoperation rates were significantly lower compared to PEEK; in the non-3D Ti subgroup, no significant differences were observed for any outcome. This finding directly reconciles the historical literature, in which comparisons of solid titanium versus PEEK yielded ambiguous conclusions (Seaman et al., 2017; Tan et al., 2021), with the contemporary literature demonstrating 3D-pTi superiority. The key insight is that it is the porosity—not the titanium chemistry—that drives the clinical advantage.

### 4.4 Cohort studies

Jacob et al. (2025) reported the largest single-center comparative cohort to date (200 patients, 277 levels) comparing 3D-pTi banana-shaped cages versus titanium-coated PEEK bullet-shaped cages in TLIF. At 1 year, the 3D-pTi group demonstrated a fusion rate of 93.3% versus 73.2% for TiPEEK (P < 0.0001), with subsidence rates of 6.0% versus 25.0%, respectively (P < 0.0001). The 24-month reoperation rate in the 3D-pTi group was 0.7% (n = 1). Importantly, the 3D-pTi group received BMP more frequently and the PEEK group more autograft, yet the fusion advantage of 3D-pTi persisted—supporting the interpretation that the porous titanium architecture provides osteoconductivity independent of, and perhaps synergistic with, biological augmentation.

Adl Amini et al. (2022a) examined fusion outcomes specifically in standalone LLIF (SA-LLIF), comparing 3D-pTi with PEEK. At early CT assessment (6–12 months), the 3D-pTi fusion rate was 95% versus 63% for PEEK; at late assessment (>12 months), the rates were 95% versus 80%. This pattern—an early, large advantage that narrows but does not disappear over time—recapitulates the “time-window effect” observed in the Duan et al. meta-analysis. Kim et al. (2022) reported similar findings in a MIS-TLIF cohort, and Yang et al. (2023) confirmed the fusion advantage of 3D-pTi at a minimum of 2 years follow-up in PLIF. Makino et al. (2022) utilized CT color mapping to demonstrate that 3D-pTi cages exhibit a distinct pattern of bone ongrowth compared to Ti-coated PEEK cages—the former characterized by three-dimensional bone penetration into the porous structure, the latter limited to two-dimensional surface attachment.

The key clinical studies are summarized in Table 2.

**TABLE 2:** Summary of key clinical studies comparing 3D-printed porous titanium and PEEK interbody cages in lumbar fusion, organized by study question and comparator.

Study	Design	Comparator	Key finding
Group A: Head-to-Head RCTs — 3D-pTi or Activated Titanium versus PEEK			
Weinberg et al. (2026)	RCT (terminated early)	3D-pTi vs PEEK	6-month BSF-3 fusion: 100% vs 0% (P < 0.001); small sample, early termination
Toop et al. (2023)	RCT	Activated Ti vs PEEK	6-month BSF-3 fusion: 84.0% vs 20.6% (P < 0.001); subsidence: 20.8% vs 41.4%

Group B: Design-Internal RCT — Porous Architecture Parameters			
Ham et al. (2024)	Multicenter RCT	Non-window vs window 3D-pTi	Fusion: 96.6% vs 93.8% (P > 0.99); porous architecture alone sufficient

Group C: Cohort Studies — 3D-pTi versus PEEK			
Jacob et al. (2025)	Retrospective cohort	3D-pTi vs PEEK	Fusion: 93.3% vs 73.2%; subsidence: 6% vs 25%
Levy et al. (2026)	Retrospective cohort	3D-pTi vs PEEK	Lower revision rate with 3D-pTi
Adl Amini et al. (2022a)	Retrospective cohort	3D-pTi vs PEEK (SA-LLIF)	Subsidence: lower with 3D-pTi
Adl Amini et al. (2022b)	Retrospective cohort	3D-pTi vs PEEK (ASD)	Early outcomes favoring 3D-pTi
Hamrick et al. (2026)	Retrospective cohort	3D-pTi vs PEEK	Subsidence: 55.4% vs 39.2% (note: higher with Ti)
Alan et al. (2023)	Propensity-matched cohort	3D-pTi vs PEEK (LLIF)	Subsidence: 8% vs 27%
Burkhard et al. (2025a)	Retrospective cohort	Pre- vs post-3D-pTi adoption	SA-LLIF utilization increased; practice pattern change
Segi et al. (2024)	Retrospective cohort	3D-pTi vs PEEK	Trabecular bone remodeling: 3-month TBR predictive
Corso et al. (2025)	Retrospective cohort	3D-pTi vs PEEK	Revision: no significant difference at early follow-up
Chahlavi (2025)	Retrospective, self-controlled (within-patient)	PEEK-Ti composite vs 3D-Ti	Subsidence: 4.8% vs 27.9%; fusion comparable

Group D: Meta-Analyses			
Wang et al. (2025)	Systematic review & MA	3D-pTi vs PEEK	19 studies; 3D-pTi advantages in early fusion and subsidence
Pairojboriboon et al. (2024)	Network MA	Multiple cage materials	SUCRA ranking: 3D-pTi (0.8) > TiPEEK (0.6) > solid Ti (0.5) > PEEK (0.3)
Liu et al. (2024)	Systematic review & MA	3D-pTi vs PEEK (LLIF)	Lower subsidence with 3D-pTi in LLIF
Duan et al. (2024)	Systematic review & MA	3D-pTi vs PEEK	Higher 6-month fusion, lower 12-month subsidence with 3D-pTi

Group E: TiPEEK versus Uncoated PEEK			
Hasegawa et al. (2020)	Multicenter RCT	TiPEEK vs PEEK (PLIF)	Better 6-month fusion with TiPEEK (OR 2.27, 95% CI 1.09–4.74)
Mariscal et al. (2025)	Systematic review & MA	TiPEEK vs PEEK	Improved fusion; effect modest and procedure-dependent
Kincaid et al. (2025)	Systematic review & MA	Ti-coated vs uncoated PEEK (cervical & lumbar)	25 RCTs; coated PEEK outperformed uncoated in PLIF and TLIF

Evidence contribution: Direct comparative evidence from randomized designs. Toop et al. used activated titanium (nano-etched surface, micro-lattice) rather than fully interconnected 3D-printed porous architecture; this distinction should be considered when comparing effect sizes between studies.

Evidence contribution: Design-internal evidence comparing two 3D-pTi cage designs. This study does not provide evidence on 3D-pTi versus PEEK., Cited as proof-of-concept that the porous titanium architecture alone supports fusion without supplemental bone graft.

Evidence contribution: Observational comparative evidence. Confounding by cage geometry (size, shape, footprint) is a recognized limitation; see Section 5.2 for detailed discussion. Hamrick et al. (2026) reported higher subsidence with 3D-pTi, and Chahlavi (2025) reported lower subsidence with a PEEK-Ti composite than with 3D-Ti; both are discussed in detail in Section 5.2 as critical counterpoints to the overall trend. The Chahlavi self-controlled (within-patient) design provides strong control of individual-level confounders, but its findings apply to the specific geometries compared and should not be generalized across material classes.

*Evidence contribution:* Aggregate evidence synthesis. Between-study heterogeneity in 3D-pTi cage design and surgical technique limits precision; see Section 10.4. No pairwise comparison in the Pairojboriboon NMA, reached statistical significance.

*Evidence contribution:* Comparative evidence for coating strategies. TiPEEK, improves upon uncoated PEEK, but does not achieve three-dimensional bone ingrowth through the implant volume (see Section 7.1).

## 5 Subsidence: Material or geometry?

### 5.1 Evidence supporting reduced subsidence with 3D-pTi

The meta-analytic evidence described above consistently reports lower subsidence rates with 3D-pTi, most robustly in the LLIF-specific analysis by Liu et al. (2024) (OR 0.25). Macki et al. (2019) reported in a systematic review that LLIF subsidence rates vary widely (3%–58%) depending on the definition, measurement method, and patient population, underscoring the need for standardized reporting. Several single-study findings corroborate the pattern of lower subsidence with 3D-pTi. Alan et al. (2023) reported, in a propensity score-matched LLIF cohort (97 vs. 97 levels), subsidence rates of 8% for 3D-pTi versus 27% for PEEK, with a number needed to treat (NNT) of 6. Adl Amini et al. (2021) reported severe subsidence (Marchi grade II–III) in only 3.0% of 3D-pTi SA-LLIF levels versus 18.5% for PEEK. Segi et al. (2023) demonstrated that vertebral endplate concavity (VEC)—a radiographic marker of endplate micro-damage—occurred in only 8% of 3D-pTi XLIF levels compared to 45% of PEEK levels, suggesting that the porous titanium architecture may be less traumatic to the endplate during impaction and more protective of endplate integrity during postoperative loading.

### 5.2 Contradictory data and the multifactorial model

Several studies challenge the prevailing narrative that 3D-pTi uniformly reduces subsidence. Hamrick et al. (2026) analyzed 130 TLIF patients (PEEK 74, titanium 56) and found that overall subsidence rates were numerically higher in the titanium group (55.4% vs. 39.2%, P = 0.067), as were high-grade subsidence rates (≥25% height loss: 51.5% vs. 45.5%, P = 0.186), although neither difference reached statistical significance. Several features of this study complicate interpretation: (i) the titanium group included a mixture of static and expandable cages, and expandable cages may generate additional endplate stress during the expansion process; (ii) titanium cages were significantly shorter (9.4 vs. 10.0 mm, P = 0.046); (iii) banana-shaped cages were more common in the PEEK group (28.6% vs. 10.9%, P = 0.016), and cage shape—independent of material—influences endplate stress distribution.

Chahlavi (2025) added a distinctive methodological perspective through a retrospective, self-controlled design in which 48 patients each received both a PEEK-titanium (PEEK-Ti) composite cage and a 3D titanium (3D-Ti) cage during the same multilevel TLIF procedure—thereby controlling, within each patient, for bone quality, comorbidities, and surgeon technique. At 6 months, the subsidence rate was significantly lower with PEEK-Ti than with 3D-Ti (4.8% versus 27.9%), while fusion rates were comparable. This finding is notable precisely because it inverts the more common pattern: here a titanium-containing composite (PEEK-Ti) outperformed a fully porous titanium cage on the subsidence endpoint. Rather than contradicting the central thesis of this review, the result reinforces it—subsidence is governed principally by geometric and structural design variables rather than by the presence of titanium *per se*. The study nonetheless carries important limitations that preclude overgeneralization: its retrospective nature, modest sample size, short (6-month) radiographic endpoint, and the comparison of specific cage geometries rather than a controlled material-only variable. Its contribution is best understood not as definitive evidence that PEEK-Ti is superior to 3D-Ti, but as a demonstration that “titanium” is not a homogeneous category, and that design-specific comparisons are required before any material class is declared superior on subsidence.

Levy et al. (2026) provided the most analytically rigorous examination of this question in a cohort of 136 patients (169 levels) undergoing TLIF with either 3D porous titanium or non-porous PEEK cages. In univariate analysis, the titanium group exhibited a significantly higher rate of moderate (≥2 mm) subsidence (77.2% vs. 32.4%, P < 0.001). However, the titanium cages in this cohort had a significantly smaller mean surface area (219 mm^2^ vs. 266 mm^2^). In multivariate logistic regression adjusting for cage surface area, cage shape, and preoperative disc height, cage material was no longer an independent predictor of moderate subsidence (OR 3.66, 95% CI 0.74–18.18, P = 0.109). This analysis strongly supports the interpretation that subsidence is primarily driven by geometric factors—especially endplate coverage area—rather than by material identity *per se*.

### 5.3 Reconciliation

These apparently contradictory findings should not be dismissed as confounded; rather, they demonstrate that subsidence is governed by geometric design variables—footprint area, shape, height, endplate preparation, and posterior instrumentation—that can override material-level differences (Campbell et al., 2020; Marchi et al., 2013; Agarwal et al., 2020; Polikeit et al., 2003). Xi et al. (2020) provided supporting evidence by demonstrating that lower vertebral Hounsfield units (a CT-based surrogate for bone mineral density) are independently associated with cage subsidence after LLIF, while Campbell et al. (2020) identified age and BMP use—rather than cage material—as the dominant predictors of subsidence in their LLIF cohort. Schreiber et al. (2011) established the methodological basis for using Hounsfield units to assess bone mineral density in spinal surgery. Wang et al. (2025) demonstrated in their meta-analysis that posterior instrumentation attenuates the effect of cage material on subsidence—a finding consistent with the notion that mechanical stabilization, whether provided by an internal fixation construct or by rapid biological fixation, can override material-related differences.

Consistent with this multifactorial model, the self-controlled data of Chahlavi (2025) further caution against treating “titanium” as a monolith: a titanium-containing composite can yield lower subsidence than a porous titanium cage when the geometric designs differ, underscoring that material identity alone does not dictate subsidence risk.

This recognition does not diminish the value of 3D-pTi; it emphasizes that optimal cage sizing and positioning are essential regardless of material choice. As noted in Section 3.2, it is critical to recognize that “3D-pTi” is not a single implant but a family of designs with substantial inter-manufacturer heterogeneity in porosity distribution, lattice architecture, pore geometry, surface modification strategies, and AM processing parameters (Su and Sun, 2026). Clinical outcomes reported for one 3D-pTi cage design should not be indiscriminately extrapolated to all designs within the material class.

## 6 Beyond radiographic fusion: reoperation and functional outcomes

### 6.1 The imaging-clinical dissociation

A recurring observation across multiple studies is the apparent dissociation between the dramatic radiographic fusion advantage of 3D-pTi and the more modest or non-significant between-group differences in patient-reported outcome measures (PROMs). Weinberg et al. (2026) reported significant within-group PROM improvements in the 3D-pTi arm at 6 months but non-significant between-group differences. Wang et al. (2025) found no significant differences in postoperative visual analog scale (VAS) back pain, VAS leg pain, or Oswestry Disability Index (ODI) scores between titanium and PEEK groups in their 19-study meta-analysis. Duan et al. (2024) reported non-significant differences in complication rates.

However, the reoperation data provide a crucial link between radiographic and clinical failure. Levy et al. (2026) reported that the overall reoperation rate was 2.6-fold higher in the PEEK group (28.6% vs. 10.9%, P = 0.026), and—most importantly—the reoperation rate specifically for pseudarthrosis was over fivefold higher (11.4% vs. 2.0%, P = 0.038), despite the absence of a significant difference in conventional Bridwell-grade radiographic fusion assessment between groups. This suggests that standard radiographic criteria—specifically the Bridwell grading system—may systematically overestimate true functional fusion in the presence of PEEK cages. As discussed in the Introduction, the “locked pseudoarthrosis” phenomenon (Fraser) can produce a CT appearance that satisfies Bridwell grade I/II criteria (peripheral bridging bone) while the bone-PEEK interface is mechanically incompetent.

Jacob et al. (2025) corroborate this interpretation: the 24-month reoperation rate in the 3D-pTi cohort was 0.7%—substantially lower than historically reported rates for PEEK TLIF. Corso et al. (2025), in their large U.S. administrative database analysis, demonstrated low cumulative reoperation and revision rates in both groups at 3 and 5 years, though trends favoring 3D-pTi emerged over time. Khan et al. (2022) reported that Tritanium posterolateral cages were associated with favorable cost-effectiveness compared to propensity-matched PEEK cages.

### 6.2 Trabecular bone remodeling: a functional fusion biomarker

Segi et al. (2024) introduced trabecular bone remodeling (TBR) as a novel CT-based biomarker of functional osseointegration after posterior lumbar interbody fusion. TBR is identified on coronal CT reconstructions as linear bands of thickened trabecular bone extending from the cage-endplate contact zone toward the pedicle screw trajectory, reflecting adaptive bone remodeling in response to stress transmission through a successfully integrated cage. In comparative analysis, 3D-pTi cages demonstrated a TBR-positive rate of 93% (38/41 successfully fused levels) versus 91% (30/33) for PEEK. The more informative finding concerned the pattern of TBR: 3D-pTi cages more frequently exhibited the coexistence of TBR and vertebral endplate cysts (VEC), interpreted as a dynamic “destruction-repair equilibrium”—localized endplate micro-damage triggering an osteogenic response that ultimately stabilizes the interface. The absence of this coexistence pattern in PEEK cages may reflect a fundamentally different (and less biomechanically adaptive) cage-endplate interaction. Tanida et al. (2016) had previously demonstrated that VEC formation is a predictor of nonunion after lumbar interbody fusion, with titanium and PEEK cages exhibiting distinct VEC patterns that correlate with fusion outcomes.

TBR and related dynamic imaging biomarkers may prove valuable in discriminating between “true osseous fusion” and “radiographically adequate but mechanically insufficient fibrous stabilization”—a distinction that existing static grading systems (Bridwell, BSF) were not designed to make.

## 7 Hybrid strategies: Can PEEK Be “activated”?

### 7.1 Titanium-coated PEEK and plasma-sprayed PEEK

In response to the biological limitations of uncoated PEEK, surface modification strategies—most prominently titanium coating—aim to confer osteoconductive surface properties while retaining the mechanical and imaging advantages of the PEEK core. Hasegawa et al. (2020) conducted the highest-quality evidence in this domain: a multicenter, prospective, randomized study of 149 patients undergoing single-level TLIF or PLIF with either TiPEEK or uncoated PEEK cages. The TiPEEK group demonstrated significantly higher bone fusion rates at 6 months (OR 2.27, 95% CI 1.09–4.74, P = 0.03), with similar subsidence rates between groups.

The meta-analysis by Mariscal et al. (2025) confirmed that TiPEEK was favored over uncoated PEEK at 6 months (RR 1.16, P = 0.009) but found no significant difference at 12 months. Kincaid et al. (2025) performed a comprehensive systematic review and meta-analysis restricted to RCTs across ACDF, PLIF, and TLIF, comparing PEEK with titanium, coated PEEK, and other materials. In lumbar procedures (PLIF and TLIF), coated PEEK tended to produce higher fusion rates than uncoated PEEK, but the meta-analysis emphasized that the effect was “modest and procedure-dependent,” with substantial heterogeneity (I^2^ = 81.6%). Critically, the majority of included RCTs had follow-up durations shorter than 24 months.

The fundamental biological limitation of coating strategies is conceptual rather than technical: as identified by Torstrick et al. (2018), who directly compared porous PEEK with plasma-sprayed titanium-coated PEEK, the porous architecture—not the titanium surface—was the dominant determinant of osseointegration quality, with porous PEEK significantly outperforming titanium-coated PEEK in bone-implant contact and biomechanical fixation. A coating, however well-applied, provides only a two-dimensional surface modification; it cannot create the three-dimensional interconnected porosity that supports vascular infiltration, osteoprogenitor cell migration, and the formation of a true bone-implant composite. This represents a fundamental biological constraint that no surface coating—titanium, hydroxyapatite, or otherwise—can transcend.

Additionally, mechanical durability concerns exist. Kienle et al. (2016) demonstrated in an *in vitro* impaction model that titanium coatings on PEEK can undergo wear and delamination during cage insertion, generating titanium particulate debris. The *in vivo* consequences of this debris—whether it triggers a clinically significant inflammatory response or merely represents a benign finding—remain uncertain. Walsh et al. (2015) demonstrated that plasma-sprayed titanium coating improves the bone-implant interface compared to uncoated PEEK in a sheep model, with significantly higher interface shear strength at 4 and 12 weeks (8.0 and 18.1 MPa vs. 1.7 and 1.8 MPa).

### 7.2 Additively manufactured porous PEEK

An alternative approach involves creating porosity within the PEEK itself via AM. Torstrick et al. (2018) demonstrated that porous PEEK significantly improved bone-implant contact and interfacial stiffness compared to titanium-coated PEEK in a rat model. Basgul et al. (2018) characterized structure-property relationships for 3D-printed PEEK intervertebral lumbar cages produced using fused filament fabrication, providing foundational data for this emerging technology. However, additively manufactured PEEK remains at an early stage of development; clinical data are virtually absent, and the mechanical properties of porous PEEK—particularly fatigue resistance under cyclic spinal loading—have not been adequately characterized for load-bearing interbody applications.

### 7.3 The SUCRA gradient: quantitative evidence of a performance hierarchy

The Pairojboriboon et al. (2024) NMA provides treatment-ranking probabilities derived from the available comparative evidence for TLIF cage materials: 3D-pTi ranked highest (SUCRA 0.8), followed by titanium-coated PEEK (0.6), solid titanium (0.5), and unmodified PEEK (0.3). SUCRA values represent the cumulative probability that a given treatment occupies each possible rank in a simulated sampling distribution, and should be interpreted as treatment-ranking probabilities rather than as a directly causal biological hierarchy or a quantitative dose–response gradient.

The observed ranking is directionally consistent with the mechanistic framework that a fully interconnected porous architecture provides the greatest biological interface for osseointegration, a surface coating provides an intermediate degree of biological engagement, and an unmodified bioinert surface provides the least. However, three important statistical caveats apply: (1) no pairwise comparison in this network meta-analysis reached statistical significance; (2) the rankings are influenced by network geometry and the precision of individual study estimates; and (3) SUCRA values should not be interpreted as quantitative measures of absolute treatment effect. The clinical implication is that while the evidence consistently suggests a ranking of fusion performance, the magnitude and certainty of the differences between adjacent ranks—particularly between 3D-pTi and TiPEEK—remain imprecisely estimated.

The three modes of bone-implant integration—surface coating, surface apposition (on-growth), and three-dimensional bone ingrowth (in-growth)—are illustrated in Figure 2.

**FIGURE 2:**
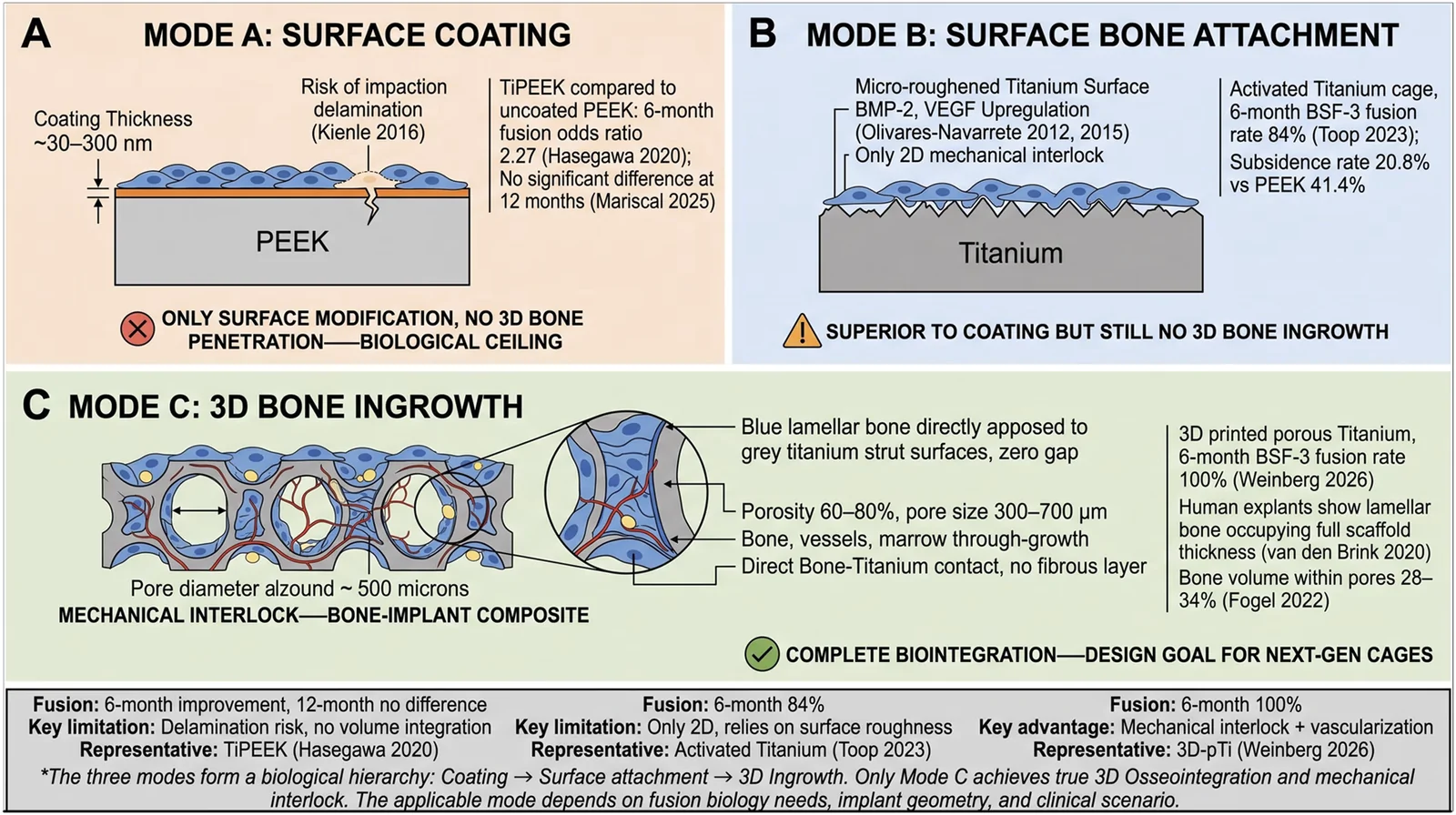
Three modes of bone-implant integration in lumbar interbody cages. **(A)** Surface coating: a thin titanium or hydroxyapatite layer (∼30–300 nm) on a solid PEEK core permits surface cell attachment only. Coating delamination during impaction has been documented (Kienle et al., 2016). Titanium-coated PEEK achieves modestly improved 6-month fusion (OR 2.27; Hasegawa et al., 2020) but no significant difference at 12 months (Mariscal et al., 2025). **(B)** Surface apposition (on-growth): micro-roughened titanium promotes osteoblast differentiation and two-dimensional bone attachment via BMP-2 and VEGF upregulation (Olivares-Navarrete et al., 2012; 2015). Activated titanium cages achieve 84.0% BSF-3 fusion at 6 months (Toop et al., 2023) but no bone penetrates the solid core—an intermediate strategy that improves on coating without achieving full three-dimensional integration. **(C)** Three-dimensional bone ingrowth (in-growth): interconnected porosity (60%–80%, 300–700 μm) supports vascular infiltration, osteoprogenitor migration, and *de novo* bone formation throughout the implant volume, creating mechanical interlock. Human retrieval confirms mature lamellar bone occupying the full scaffold height in direct contact with titanium struts (van den Brink and Lamerigts, 2020). 3D-pTi achieves 100% BSF-3 fusion at 6 months (Weinberg et al., 2026) with bone occupying 28%–34% of pore volume (Fogel et al., 2022b). Design-internal evidence confirms that the porous architecture alone—without a graft chamber—is sufficient for fusion (Ham et al., 2024). The three modes form a biological hierarchy—coating → on-growth → in-growth—with only Mode C achieving true three-dimensional osseointegration. Created with BioRender.

## 8 PEEK in contemporary practice: enduring indications

The preceding sections have detailed the biological rationale and clinical evidence supporting the porous architecture strategy embodied by 3D-pTi. However, a balanced assessment requires a candid and detailed examination of the specific advantages that PEEK retains in contemporary practice. These advantages are not merely historical artifacts; they represent clinically meaningful benefits in defined patient populations and surgical scenarios.

### 8.1 Radiolucency: more than convenience

PEEK’s radiolucency is not merely a convenience for the surgeon; it has genuine implications for long-term patient management. Titanium implants produce metal artifacts on CT and magnetic resonance imaging (MRI) that can impede evaluation of the interbody fusion mass and, critically, of adjacent segment degeneration. Although Carter et al. (2020) demonstrated that additively manufactured porous Ti-6Al-4V structures produce substantially reduced MRI susceptibility artifacts compared to solid titanium—owing to the reduced metal volume fraction—titanium artifacts remain greater than those from radiolucent PEEK. For younger patients (under approximately 50 years) who will require decades of surveillance for adjacent segment pathology, the unobstructed visualization provided by PEEK may be clinically meaningful. The Bridwell and BSF grading systems were originally developed and validated for radiolucent interbody implants, and their application to metallic cages with varying degrees of radiographic artifact remains an under-recognized source of assessment variability. The clinical significance of artifact-related differences for serial monitoring has not been formally quantified in comparative studies and represents an important evidence gap.

### 8.2 Revisability: when fibrous encapsulation is an advantage

The biological property that compromises fusion with PEEK—fibrous encapsulation—becomes a tangible advantage in specific revision scenarios. As demonstrated by van den Brink and Lamerigts (2020), a well-integrated 3D-pTi cage may require a chisel for removal, with the attendant risks of endplate fracture, dural tear, or nerve root injury. Levy et al. (2026) noted that in all reoperations for pseudarthrosis in their cohort, the existing cage was retained *in situ* regardless of material, and the revision consisted solely of supplemental posterior instrumentation and posterolateral fusion. This strategy is particularly suited to 3D-pTi (where the cage is already osseointegrated and removal would be morbid).

When future reoperation involving cage removal is specifically anticipated—such as cases of established deep infection requiring eventual implant exchange, symptomatic cage malposition or migration, implant-related neural compression, or planned staged reconstruction requiring cage exchange—the fibrous encapsulation characteristic of PEEK cages may facilitate safer explantation compared with the osseointegrated interface of 3D-pTi cages. Retrieval histology confirms that 3D-pTi cages achieve direct bone–implant contact without an intervening fibrous layer (van den Brink and Lamerigts, 2020), which complicates safe removal and may increase the risk of endplate fracture, dural tear, or nerve root injury during explantation.

It should be noted, however, that in most surgeries for adjacent-segment disease, the prior interbody cage is not removed; the procedure involves extension of the construct to the newly degenerated adjacent level. Therefore, anticipated adjacent-segment disease alone does not constitute a strong indication for PEEK on the basis of revisability. The clinical scenario in which PEEK’s ease of removal offers a material advantage is limited to the specific circumstances enumerated above.

### 8.3 Clinical track record

PEEK cages have been implanted in millions of patients worldwide over more than 25 years. The long-term safety profile—including infection rates, wear characteristics, absence of systemic effects, and compatibility with multiple sterilization methods—is extensively characterized. 3D-pTi cages, in contrast, have been in clinical use for less than a decade. The long-term risks specific to the porous titanium architecture have not been systematically characterized beyond the 5-year horizon. These include: late hematogenous infection in the high-surface-area porous structure (theoretical concern, not documented); metal ion release (titanium, aluminum, vanadium) with potential systemic accumulation; and particulate wear debris from micromotion at strut junctions within the lattice. For low-risk patients—young, normal bone quality, primary single-level fusion—the mature safety record of PEEK may represent the more prudent choice, even if the biological properties of 3D-pTi are theoretically superior.

### 8.4 Cost and accessibility

The cost-effectiveness analysis by Alan et al. (2023) was performed in a specific institutional setting where procurement costs for the two cage types were equivalent, rendering 3D-pTi automatically dominant (lower subsidence, lower reoperation, same cost). Sensitivity analysis was required to identify the price threshold at which 3D-pTi would cease to be cost-effective under different market conditions. In many healthcare systems, PEEK cages remain substantially less expensive than 3D-pTi cages. For patients at low baseline risk of pseudarthrosis or subsidence—primary single-level fusion in a healthy patient with normal bone quality—the incremental implant cost of 3D-pTi may not be offset by reoperation avoidance. Malone et al. (2022) concluded that bioactive interbody devices may reduce the overall cost burden of achieving lateral lumbar fusion primarily through reoperation avoidance, but this conclusion depends heavily on institutional reoperation rates and the cost differential between cage types. Institution-specific cost-effectiveness analyses incorporating local procurement costs, reoperation rates, and payer mix are required for evidence-based resource allocation. Generalizable conclusions about the cost-effectiveness of one material over the other cannot be drawn from the currently available data.

### 8.5 Titanium-coated PEEK: a valid intermediate

As discussed in Section 7.1, titanium-coated PEEK represents a compromise strategy that improves early fusion relative to uncoated PEEK (OR 2.27 at 6 months; Hasegawa et al., 2020; Mariscal et al., 2025) while preserving the radiolucency and well-characterized safety profile of the PEEK core. For surgeons who wish to improve upon the biological performance of uncoated PEEK without transitioning to a fully porous metallic implant with its associated imaging artifacts and revisability challenges, TiPEEK offers an evidence-supported intermediate option. The limitation of this strategy—the biological constraint imposed by the solid core that prevents three-dimensional bone ingrowth—is fundamental but may be clinically acceptable in lower-risk scenarios where the incremental benefit of full osseointegration does not justify the trade-offs of a fully porous metallic cage.

## 9 Clinical decision framework

The incorporation of cage material as a key variable in structured decision-making pathways for SA-LLIF candidacy has been proposed by Adl Amini et al. (2022c), reflecting the increasing recognition that patient-specific factors and implant characteristics must be jointly considered.

### 9.1 Scenarios where 3D-pTi may be preferred (emerging indications)

For patients at elevated risk of pseudarthrosis—including revision surgery for failed fusion, multilevel constructs (particularly beyond three levels), smokers, and patients with conditions that impair bone healing—the accelerated early fusion and osteoconductive surface of 3D-pTi provide a biologically plausible advantage. The Levy et al. (2026) finding of a fivefold reduction in pseudarthrosis-related reoperation (2.0% vs. 11.4%), despite equivalent conventional radiographic fusion grades, is particularly relevant in this context. However, it should be emphasized that this evidence derives from retrospective cohort data, not from prospective randomized comparisons conducted specifically in high-risk populations.

In standalone LLIF, where the cage must provide anterior column support without the stabilizing effect of posterior instrumentation, lower subsidence rates have been observed with 3D-pTi in multiple cohort studies and meta-analyses (Liu et al., 2024; Alan et al., 2023; Adl Amini et al., 2021), supporting its preferential use. The Burkhard et al. (2025a) 10-year trend data, demonstrating a significant expansion of SA-LLIF utilization coincident with institutional adoption of 3D-pTi (from 28.3% to 44.8%, P < 0.001), provide real-world corroboration, though these observational data cannot establish causality. Burkhard et al. (2025b) also reported, in a separate analysis of risk factors for revision surgery, that 3D-pTi SA-LLIF was associated with a greater than 50% reduction in the hazard of revision compared to PEEK. Adl Amini et al. (2022b) further extended this evidence base to the clinically challenging scenario of symptomatic adjacent segment degeneration, demonstrating favorable early outcomes with 3D-pTi cages in SA-LLIF for this indication.

For patients with compromised bone quality (osteoporosis, osteopenia), the available evidence requires careful stratification by the nature of the supporting data:

#### 9.1.1 Observation

Burkhard et al. (2025a) reported that the proportion of standalone LLIF procedures utilizing 3D-pTi cages in patients with osteoporosis increased from 14.3% in the pre-3D-pTi era to 45.5% after institutional adoption of 3D-pTi cages (P = 0.012). This observation reflects evolving practice patterns but does not constitute evidence of superior clinical outcomes in this subgroup. No randomized controlled trial has directly compared 3D-pTi and PEEK cages in a population stratified by bone mineral density.

#### 9.1.2 Hypothesis

A mechanistically plausible rationale exists for potential advantages of 3D-pTi in osteoporotic bone: the microporous endplate interface may distribute axial load more homogeneously across the structurally compromised endplate, and progressive bone ingrowth may provide time-dependent biological stabilization that compensates for impaired primary mechanical fixation. Preclinical data from a dental implant model in osteoporotic beagle dogs suggest that a porous titanium surface can enhance osseointegration via bone ingrowth into the pore space under conditions of compromised bone quality (Ko et al., 2021); however, the model (mandibular dental implant, apical surface modification) differs from the fully interconnected porous architecture and mechanically loaded interbody environment addressed in this review, and the hypothesis has not been tested in a dedicated prospective comparative clinical study of lumbar interbody fusion.

#### 9.1.3 Recommendation

For patients with compromised bone quality, the use of 3D-pTi cages represents an emerging but unvalidated indication. Cage selection should be individualized based on endplate morphology, bone quality as assessed by preoperative CT Hounsfield units, and the mechanical demands of the intended construct. Clinicians should not extrapolate from utilization trends to efficacy claims, and any recommendation in favor of 3D-pTi in this population is provisional pending prospective, bone-density-stratified comparative evidence.

### 9.2 Scenarios where PEEK or TiPEEK remain reasonable choices

For primary single-level fusion in patients with normal bone quality, the clinical significance of accelerated early fusion—the most consistent advantage of 3D-pTi—has not been demonstrated to translate into superior long-term patient-reported outcomes (Wang et al., 2025; Duan et al., 2024). In this common clinical scenario, PEEK’s radiolucency, well-characterized safety record spanning more than two decades, and lower cost in many healthcare systems make it a reasonable first-line choice. TiPEEK provides an intermediate option with improved early fusion biology while retaining the core advantages of PEEK (Hasegawa et al., 2020; Mariscal et al., 2025).

For young patients (under approximately 50 years) who will require decades of MRI surveillance for adjacent segment degeneration, the unobstructed imaging provided by PEEK or TiPEEK may be clinically preferred, although the magnitude of this advantage has not been formally quantified.

When future reoperation with cage removal is anticipated—cases of established deep infection requiring eventual implant exchange, symptomatic cage malposition or migration, implant-related neural compression, or planned staged reconstruction requiring cage exchange—PEEK’s fibrous encapsulation facilitates safer explantation. As noted in Section 8.2, anticipated adjacent-segment disease alone does not constitute a strong indication for PEEK on the basis of revisability, as most adjacent-segment surgeries do not involve removal of the prior cage.

### 9.3 Scenarios where evidence is insufficient

Direct comparative evidence is sparse or absent for several clinically important scenarios. No dedicated RCT has compared 3D-pTi and PEEK in osteoporotic patients. No prospective studies have compared the two materials in revision surgery requiring cage exchange. Multilevel fusion beyond three levels has limited comparative data. Patients with active or recent spinal infection have not been studied. In these scenarios, cage selection should be individualized, and any clinical recommendation should be acknowledged as provisional, pending prospective evidence.

A clinical decision framework integrating the evidence across these scenarios is provided in Table 3.

**TABLE 3:** Current evidence for cage selection across clinical scenarios.

Clinical scenario	Evidence source	3D-pTi considerations	PEEK/TiPEEK considerations	Key references
Primary single-level, normal BMD	RCT evidence (Weinberg et al., 2026; Toop et al., 2023) + meta-analyses (Wang et al., 2025; Duan et al., 2024)	Accelerated early fusion demonstrated in RCTs; long-term PROM benefit unproven	25-year safety record; radiolucency; lower cost in most healthcare systems	Weinberg et al. (2026), Toop et al. (2023), Wang et al. (2025), Duan et al. (2024)
Osteoporosis	Utilization/trend data only; no direct comparative evidence	Theoretical endplate protection via microporous load distribution; progressive biological stabilization plausible but unproven. Surgeon adoption trends (Burkhard et al., 2025a) constitute practice-pattern data, not efficacy evidence. No dedicated RCT in osteoporotic population. Preclinical data in osteoporotic animal model suggest enhanced osseointegration (Ko et al., 2021) but untested clinically	Long clinical experience; geometric optimization (maximizing footprint area) essential regardless of material	Burkhard et al. (2025a), Burkhard et al. (2025b), Xi et al. (2020), Schreiber et al. (2011), Ko et al. (2021) (dental implant model)
Standalone LLIF	Retrospective cohort studies (Alan et al., 2023; Adl Amini et al., 2021; Burkhard et al., 2025a) + one focused meta-analysis (Liu et al., 2024)	Lower subsidence in multiple cohort studies and one focused meta-analysis (Liu et al., 2024, OR 0.25). Expanded SA-LLIF adoption temporally associated with institutional transition to 3D-pTi (Burkhard et al., 2025a)—observational, not causal. Burkhard et al. (2025b) also reported >50% reduction in revision hazard with 3D-pTi in SA-LLIF.	TiPEEK offers intermediate risk profile if metal artifact or revisability concerns predominate	Liu et al. (2024), Alan et al. (2023), Adl Amini et al. (2021), Burkhard et al. (2025a), Burkhard et al. (2025b)
Revision for pseudarthrosis	Retrospective cohort only (Levy et al., 2026)	May achieve fusion where PEEK failed (Levy et al., 2026: 5-fold reduction in pseudarthrosis reoperation). Removal challenging if osseointegrated—chisel required (van den Brink and Lamerights, 2020)	Easier removal if cage exchange needed; fibrous encapsulation facilitates atraumatic explantation	Levy et al. (2026), van den Brink and Lamerights (2020)
Young patient (<50 yr)	No direct comparative evidence	Metal artifact on MRI may impede decades of adjacent-segment surveillance (though porous AM structures reduce artifact vs. solid Ti; Carter et al., 2020)	Radiolucency critical for unobstructed long-term monitoring of adjacent segments. Magnitude of artifact-related clinical advantage not formally quantified	Carter et al. (2020)
Multilevel fusion (≥3 levels)	Limited comparative data (Jacob et al., 2025)	Theoretical advantage for achieving fusion at all instrumented levels; limited comparative data	Established safety profile in multilevel constructs	Jacob et al. (2025)
Anticipated future cage removal	No direct comparative evidence	Bone ingrowth complicates safe explantation—endplate fracture, dural tear, nerve injury risk (van den Brink and Lamerights, 2020). Anticipated adjacent-segment disease alone does not constitute a strong indication for PEEK on revisability grounds, as most ASD surgeries do not involve cage removal (see Section 8.2)	Fibrous encapsulation facilitates safer removal; clinically relevant when infection, malposition, or planned cage exchange is anticipated	van den Brink and Lamerights (2020)
Cost-constrained setting	Institution-dependent cost analyses (Alan et al., 2023; Khan et al., 2022; Malone et al., 2022)	May reduce reoperation-related costs in high-risk patients (NNT = 6 for subsidence avoidance; Alan et al., 2023). Cost-effectiveness threshold identified by sensitivity analysis	Lower upfront implant cost in most healthcare systems; cost-effective in low-risk scenarios where reoperation probability is low	Alan et al. (2023), Khan et al. (2022), Malone et al. (2022)
Adjacent segment degeneration (SA-LLIF)	Single retrospective cohort (Adl Amini et al., 2022b)	Favorable early outcomes with 3D-pTi cages reported (Adl Amini et al., 2022b). Lower severe subsidence vs PEEK.	TiPEEK may be a reasonable alternative when radiolucency is prioritized	Adl Amini et al. (2022b)

Abbreviations: ASD, adjacent segment disease; BMD, bone mineral density; NNT, number needed to treat; PROM, patient-reported outcome measure; SA, standalone; TiPEEK, titanium-coated PEEK.

## 10 Health economics and unresolved concerns

### 10.1 Cost-effectiveness

The economic dimension of cage material selection is institution- and health-system-dependent. Alan et al. (2023) performed the most rigorous cost analysis to date in a propensity score-matched LLIF cohort (97 vs. 97 levels). At their institution, procurement costs for the two cage types were equivalent, rendering 3D-pTi automatically dominant (lower subsidence, lower reoperation, same cost). Sensitivity analysis determined the price threshold at which 3D-pTi would cease to be cost-effective, and the NNT was 6. Malone et al. (2022) concluded that bioactive interbody devices may reduce the overall cost burden of achieving lateral lumbar fusion primarily through reoperation avoidance. However, Kincaid et al. (2025) noted that in some healthcare settings, overall hospital costs for PEEK-based fusions have been slightly lower than for titanium-based fusions. Khan et al. (2022) reported favorable cost-effectiveness for Tritanium posterolateral cages versus PEEK in propensity-matched analysis. The generalizability of cost-effectiveness conclusions is therefore limited, and institution-specific analyses incorporating local procurement costs, institutional reoperation rates, and payer mix are required.

### 10.2 Imaging artifacts

As discussed in Section 8.1, titanium implants produce metal artifacts on CT and MRI. Carter et al. (2020) demonstrated that porous AM structures produce substantially reduced artifacts compared to solid titanium. However, even with this reduction, titanium artifacts remain greater than those from radiolucent PEEK, and the clinical significance of this difference for serial surveillance of adjacent segment degeneration has not been quantified.

### 10.3 Infection risk

The high specific surface area of porous titanium has prompted theoretical concern about biofilm formation and late hematogenous infection. Levy et al. (2026) reported infection rates of 0.80% (titanium) versus 2.27% (PEEK) (P = 0.454), providing no signal of increased infection risk, though the sample size was inadequate to detect rare events. No systematic comparison of infection rates between 3D-pTi and PEEK cages has been published; this remains a theoretical concern rather than a documented risk.

### 10.4 Design heterogeneity

As noted throughout this review, “3D-pTi” encompasses a family of implants with widely varying porosity, pore size, lattice topology, surface roughness, and manufacturing parameters. Extrapolating the findings from one design to another is scientifically unjustified without confirmatory data. This heterogeneity complicates meta-analytic pooling and may explain some of the between-study variance in outcomes. Reporting standards for AM implant studies should include porosity fraction, mean pore size, lattice topology, surface roughness parameters, and manufacturing method to enable more granular between-study comparisons.

## 11 Discussion

### 11.1 Synthesis of the evidence

The translational chain of evidence examining 3D-pTi spans *in vitro* surface science (Olivares-Navarrete et al., 2012; 2015), mechanistic animal experiments (Torstrick et al., 2020; McGilvray et al., 2018; Laratta et al., 2022; Fogel et al., 2022b), human retrieval histology (van den Brink and Lamerigts, 2020), randomized controlled trials (Weinberg et al., 2026; Toop et al., 2023), design-internal RCT evidence (Ham et al., 2024), large-scale real-world data (Levy et al., 2026; Jacob et al., 2025; Burkhard et al., 2025a; Burkhard et al., 2025b), and four independent meta-analytic syntheses (Duan et al., 2024; Liu et al., 2024; Pairojboriboon et al., 2024; Wang et al., 2025). The consistency of the signal across these methodologically diverse study types—all pointing toward accelerated early fusion—is a compelling argument in favor of 3D-pTi’s clinical adoption for scenarios in which rapid and reliable biological fixation is a priority.

However, it is essential to distinguish the strength of evidence for different endpoints. The evidence for accelerated early fusion is substantially more robust than the evidence for superior functional outcomes. Meta-analyses consistently report no significant between-group differences in VAS back pain, VAS leg pain, or ODI scores at final follow-up (Wang et al., 2025; Duan et al., 2024), and the translation of earlier radiographic fusion into clinically meaningful patient benefit has not been demonstrated.

The biological mechanism underlying the fusion advantage is now well characterized: the porous architecture—rather than the titanium chemistry—is the predominant driver of osseointegration under the experimental conditions tested to date, as demonstrated by Torstrick et al. (2020) in a rat tibial model and corroborated by large-animal interbody data (Fogel et al., 2022b). This principle explains why 3D-pTi outperforms both PEEK (lacks porosity) and TiPEEK (provides favorable surface chemistry but lacks three-dimensional porosity for bone ingrowth). It also explains the subgroup effect identified by Wang et al. (2025): solid titanium—which offers favorable chemistry without porosity—does not outperform PEEK, whereas 3D-pTi—which offers both—does.

### 11.2 Different schools of thought and controversies

Two interpretive frameworks coexist in the current literature. The “material-advantage” school emphasizes the RCT and meta-analytic data and argues that 3D-pTi represents a categorical improvement over PEEK for LIF. The “geometric-primacy” school emphasizes the Levy et al. (2026) and Campbell et al. (2020) data—demonstrating that cage surface area, shape, and endplate preparation are more powerful predictors of subsidence than material—and argues that optimizing geometric design variables in a PEEK cage may yield outcomes equivalent to a suboptimally sized 3D-pTi cage.

A synthetic interpretation is that these frameworks address different clinical endpoints. For fusion biology, material and porosity are the dominant variables because they directly govern the osteoconductive microenvironment. For subsidence, geometry is the dominant variable in the early postoperative period (before meaningful bone ingrowth has occurred), while porosity becomes increasingly important over time as biological fixation supplements or replaces mechanical interlock. The clinical implication is that optimal outcomes require both a biologically active material and optimal geometric design. Neither alone is sufficient, and the two strategies are synergistic rather than mutually exclusive.

### 11.3 Fundamental issues and problems

Several fundamental issues emerge from this review. First, the definition and assessment of “fusion” remain inadequately standardized. The Bridwell and BSF grading systems were developed for allograft and autograft constructs, not for porous metallic implants that support bone through the cage rather than around it. The locked pseudoarthrosis phenomenon (Weinberg et al., 2026) implies that existing radiographic criteria may be insufficiently sensitive to discriminate between mechanically competent and incompetent fusion in PEEK constructs. The TBR concept (Segi et al., 2024) represents a promising step toward functional fusion assessment, but validation in large, prospective cohorts is lacking.

Second, the heterogeneity of “3D-pTi” as a category is both a scientific problem (complicating between-study comparisons and meta-analytic pooling) and an opportunity (the design space is large and only partially explored). Reporting standards for AM implant studies should be established to include porosity fraction, mean pore size, lattice topology, surface roughness parameters, and manufacturing method.

Third, the existing evidence is concentrated in relatively healthy, non-osteoporotic, single-level fusion populations, yet the clinical scenarios in which cage material choice is most consequential—osteoporosis, revision, multilevel constructs, deformity—are those with the least data. This “inverse evidence law” represents the most critical gap in the current literature.

### 11.4 Research gaps

The following research gaps have been identified through this review: (1) large-scale, multicenter RCTs comparing 3D-pTi and PEEK in osteoporotic patients, with bone mineral density-stratified randomization and powered for subsidence and reoperation endpoints; (2) long-term (>5-year) follow-up data from existing cohorts to assess adjacent segment disease rates, late reoperations, and durability of fusion; (3) standardized definitions and assessment protocols for fusion and subsidence, ideally incorporating dynamic biomarkers such as TBR and quantitative CT measures of bone density within the fusion mass; (4) retrieval studies systematically comparing the histology and microbiology of explanted 3D-pTi and PEEK cages from revision procedures; (5) cost-effectiveness analyses using multi-institutional data across different healthcare systems and procurement models; (6) comparative studies of different 3D-pTi designs to determine whether specific architectural parameters (pore size, porosity, lattice type) correlate with clinical outcomes.

### 11.5 Future developments

Several technological trajectories are poised to influence the field. Patient-specific 3D-pTi cages, designed from preoperative CT data to match individual endplate anatomy and optimize contact area, are in early clinical investigation (Smit et al., 2024). Bioactive surface functionalization—incorporating BMP-2, VEGF, or biomimetic peptides into the porous titanium scaffold via sustained-release microspheres (Liu et al., 2022)—may further accelerate fusion, particularly in biologically compromised hosts, though dose optimization, release kinetics, and regulatory pathways remain unresolved. Additively manufactured porous PEEK and 3D-printed tantalum (Liang et al., 2025) represent alternative materials that may compete for specific niches. Cottrill et al. (2020) performed a cross-disciplinary systematic review demonstrating that bioactive glasses have shown promise in enhancing spinal fusion in preclinical models, though clinical data remain limited. Biodegradable cages (polycaprolactone/β-tricalcium phosphate composites) have demonstrated 95.2% 12-month fusion in a small prospective series (Liu et al., 2023) and represent a conceptually attractive long-term solution that eliminates the permanent implant and its associated late complications. However, the historical failure of early bioabsorbable cages (PLDLLA), which exhibited osteolysis and high nonunion rates (Smith et al., 2010; Laubach et al., 2022), counsels caution.

### 11.6 Limitations

This review has several limitations. It is a narrative, not a systematic, synthesis. A structured systematic literature search was conducted, but formal quality assessment of individual studies and quantitative pooling were not performed. The conclusions are based on qualitative evidence evaluation and are subject to the inherent biases of narrative interpretation. The review is restricted to English-language publications, and relevant data published in other languages may have been missed. The heterogeneity of 3D-pTi cage designs, surgical techniques, graft materials, and outcome definitions across the included studies limits the precision of cross-study comparisons.

## 12 Conclusion

The two materials represent different strategies for optimizing the mechanical, biological, imaging, and economic trade-offs inherent in lumbar interbody fusion. 3D-pTi cages, through their interconnected porous architecture, can accelerate early radiographic fusion via three-dimensional bone ingrowth—a mode of biological fixation that surface-coated PEEK cannot replicate. The biological mechanism is grounded in experimental evidence that surface topography, rather than material chemistry, is the predominant driver of osseointegration quality under the conditions tested to date. Clinical evidence consistently supports accelerated early fusion, and emerging data suggest reduced pseudarthrosis-related reoperation in selected populations. However, the clinical significance of accelerated early fusion for long-term patient-reported outcomes remains to be established—meta-analyses consistently report no significant between-group differences in pain or disability scores—and cage subsidence is governed primarily by geometric factors (surface area, shape, and endplate preparation) that can override material-level differences.

PEEK retains specific and enduring advantages: radiolucency for unobstructed long-term surveillance of the fusion mass and adjacent segments, a well-characterized safety record spanning more than two decades, and greater ease of revision when cage removal is anticipated. Titanium-coated PEEK represents a valid intermediate strategy, improving early fusion over uncoated PEEK (Hasegawa et al., 2020; Mariscal et al., 2025) while preserving these advantages, though it cannot overcome the biological constraint imposed by the solid core.

The clinical question is not which material is “better,” but which trade-off profile best matches the individual patient’s needs. For primary single-level fusion in patients with normal bone quality, either material is acceptable, and the choice may be guided by surgeon preference, institutional factors, and cost. For scenarios demanding rapid and reliable biological fixation—revision for pseudarthrosis, multilevel constructs, standalone anterior column support—3D-pTi offers a biologically plausible and increasingly evidence-supported advantage. For young patients requiring decades of MRI surveillance, or those in whom future cage removal is anticipated, PEEK or TiPEEK remain rational choices. In osteoporotic and revision populations, direct comparative evidence is insufficient to mandate one material over the other, and cage selection should be individualized, with explicit acknowledgment of the uncertainty.

As longer-term data and dedicated trials in high-risk populations accumulate, the boundaries of these recommendations will require recalibration. This iterative refinement—not the declaration of a “winner”—is the appropriate trajectory for evidence-based cage material selection.
